# Outside Mainstream Electronic Databases: Review of Studies Conducted in the USSR and Post-Soviet Countries on Electric Current-Assisted Consolidation of Powder Materials

**DOI:** 10.3390/ma6104375

**Published:** 2013-09-30

**Authors:** Eugene A. Olevsky, Elena V. Aleksandrova, Alexandra M. Ilyina, Dina V. Dudina, Alexander N. Novoselov, Kirill Y. Pelve, Eugene G. Grigoryev

**Affiliations:** 1 Key Laboratory for Electromagnetic Field Assisted Processing of Novel Materials, Moscow Engineering Physics Institute, Kashirskoe Sh. 31, Moscow 115409, Russia; E-Mails: alexsandrovaev@gmail.com (E.V.A.); ilyina_am@mail.ru (A.M.I.); dina1807@gmail.com (D.V.D.); a.n.novoselov@mail.ru (A.N.N.); kpelve@gmail.com (K.Y.P.); eggrigoryev@mephi.ru (E.G.G.); 2 Powder Technology Laboratory, San Diego State University, 5500 Campanile Dr., San Diego, CA 92182, USA

**Keywords:** spark plasma sintering, electric discharge sintering, consolidation, powder materials

## Abstract

This paper reviews research articles published in the former USSR and post-soviet countries on the consolidation of powder materials using electric current that passes through the powder sample and/or a conductive die-punch set-up. Having been published in Russian, many of the reviewed papers are not included in the mainstream electronic databases of the scientific articles and thus are not known to the scientific community. The present review is aimed at filling this information gap. In the paper, the electric current-assisted sintering techniques based on high- and low-voltage approaches are presented. The main results of the theoretical modeling of the processes of electromagnetic field-assisted consolidation of powder materials are discussed. Sintering experiments and related equipment are described and the major experimental results are analyzed. Sintering conditions required to achieve the desired properties of the sintered materials are provided for selected material systems. Tooling materials used in the electric current-assisted consolidation set-ups are also described.

## 1. Introduction

Bulk materials can be produced from powders by various consolidation methods, some of which use electric current directly passing through the powder sample and/or through conductive tooling. Theoretical and applied research in this area is carried out worldwide. A significant contribution to the development of electric current-assisted consolidation has been made by scientists of the USSR and post-soviet countries; research mostly being done with the help of unique custom-made facilities. Having been published in Russian, many of the papers describing the results of these investigations are not included in the mainstream electronic databases of the scientific articles and thus are not known to the scientific community. The present review is aimed at filling this information gap.

**Figure 1:**
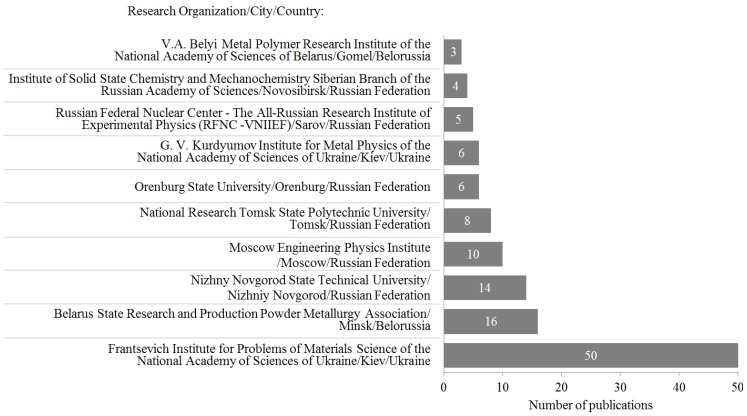
Research organizations that significantly contributed to the development of electric current-assisted consolidation of powder materials in USSR and Post-Soviet Countries.

The largest number of papers published by scientists of the USSR and post-soviet countries were written by scientists of Frantsevich Institute for Problems of Materials Science, Ukrainian Academy of Sciences—approximately 50 papers. Belarus State Research and Production Powder Metallurgy Association (Minsk) has published 20 papers. The third by the number of publications is Nizhny Novgorod State Technical University. As it seen from Figure 1, other research organizations have published up to 10 papers.

**Figure 2:**
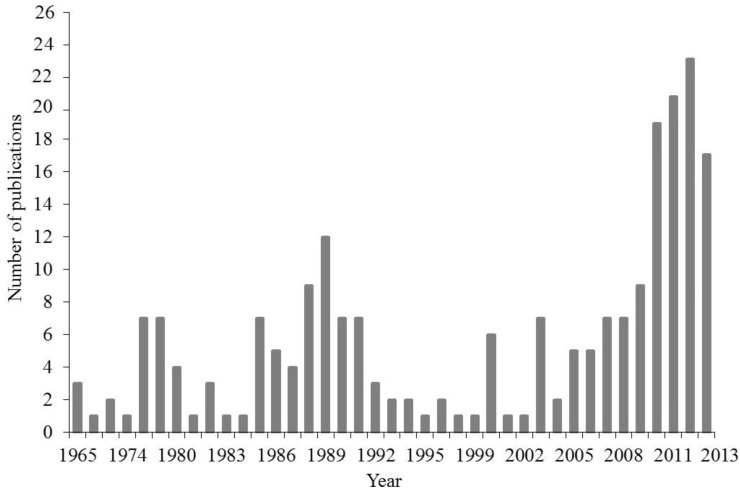
The number of articles on electric current-assisted consolidation published since 1965 in the former USSR and post-soviet countries.

Figure 2 shows the number of articles on electric current-assisted sintering of powder materials published in the former USSR and post-soviet countries in the period from 1965 to 2011. Papers published before 1981 were mainly on electric pulse sintering. The period from 1985 to 1986 revealed interest in low-voltage consolidation while electric pulse sintering drew attention again in 1987–1991. Research publications of the recent years mainly focus on low-voltage spark plasma sintering (SPS).

Although electric current-assisted sintering had already been known by the end of the 19th century, it was only in 1930s that the research in this area was begun in the USSR [1]. The first publications appeared a decade later—in the 1940s—and an intensive development of this area took place in the middle of the 1960s. Theoretical aspects of electric current-assisted sintering were developed by Raichenko [2], whose works were based on sintering models developed by Geguzin [3].

Sintering of fine-grained materials from powders is a challenging task due to rapid grain growth at the final sintering stage enabled by thermally activated diffusion. In conventional sintering methods, the processes responsible for grain growth and densification are difficult to separate due to the small difference in the activation energies of the mass transport [4]. When electric current is involved in the sintering processes, the two processes can be separated.

The technologically easiest way to consolidate conductive powders by means of electric current is to let a high current of low voltage pass directly through a pre-pressed material. For this reason, the first experiments on electric current-assisted consolidation were conducted under these described conditions. At the inter-particle contacts, local heating can take place leading to melting and sintering of the powder particles. Later on, pulsed current was shown to be more efficient in facilitating sintering [5]. The possibility of making layers on the surface of the sintered samples with peculiar characteristics was also acknowledged [5]. This sintering method along with electric discharge sintering has been studied by Raichenko and his colleagues of Frantsevich Institute for Problems of Materials Science, National Academy of Sciences of Ukraine since the beginning of the 1970s. At the same time, Bryansk Branch of the All-Union Design and Technological Institute of Construction and Road Engineering Industry in collaboration with Frantsevich Institute for Problems of Materials Science conducted research on electric pulse sintering with Rymorov [6] as team leader. In a few years, in the 1980s, investigations in this area started in the Moscow Engineering Physics Institute and were led by Balankin [7,8,9,10,11]. The research team of the Institute of Powder Metallurgy, Belarus Republic (part of USSR at that time) made also significant contribution to the development of electric current-assisted sintering and fundamental understanding of the sintering mechanisms [12]. In Nizhny Novgorod State Technical University, from 1986 till the present, electric pulse consolidation has been studied including the processes of sintering during powder rolling performed by Mal’tsev [13,14,15,16,17,18,19,20].

Electric discharge sintering belongs to a group of low-voltage sintering techniques. In the literature, it is also referred to as spark plasma sintering. This method uses short pulses of high current passing through a conductive powder or through a conductive die in the case of a non-conducting powder. The pressure is simultaneously applied to the material. It becomes possible to achieve densification in a short time and obtain an almost fully dense material [21].

Some authors, however, use the term “electric discharge sintering” to refer to a two-stage process [22]. At the first stage, the powder is shaped into a compact by a high-voltage pulse. The second stage, during which electric current of high density is applied, can last several tens of minutes. During the first stage, even in powders having an oxide film on the particle surface, the inter-particle contacts successfully form, promoting efficient sintering at the second stage. A possibility exists to find parameters of the first sintering stage such that complete sintering will occur making the second one unnecessary.

In some publications, the first stage in electric discharge sintering was associated with the formation of spark plasma between the particles induced by a high-voltage electric pulse [23]. A high energy density in the contact area leads to local temperatures as high as 103 K, which is beneficial for sintering of ceramics and refractory metals into bulk dense bodies of uniform microstructure. This allows the sintering temperatures to be reduced, which is especially important for oxide refractory materials [24].

Raichenko and his team at Frantsevich Institute for Problems of Materials Science, National Academy of Sciences of Ukraine started developing the method of electric discharge sintering in the beginning of the 1970s [5]. In the following years, the Laboratory acquired a leading position in this area. Electric discharge sintering was also the subject of research at National Research Tomsk State Polytechnic University, Russian Federation [25,26].

**Figure 3:**
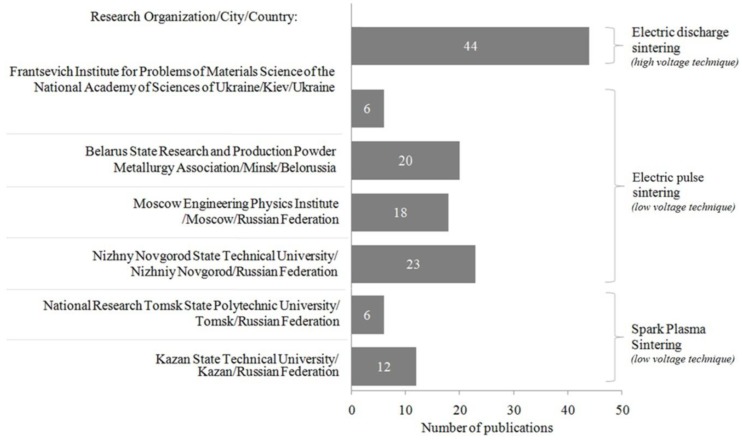
Publications distributed over different research organizations and methods of electric current-assisted consolidation.

The distribution of publications over different research organizations and methods of electric current-assisted consolidation are presented in Figure 3. The method of consolidation that received the most attention was electric pulse sintering, which was studied by the largest number of research groups.

## 2. Methods of Electric Current-Assisted Consolidation of Powder Materials and Related Equipment

This chapter reviews the methods of electric current-assisted consolidation and the corresponding equipment. The first part of the chapter deals with electric pulse sintering methods, which use a single pulse of electric current of high voltage obtained from a discharge of a capacitor bank. The second part of the chapter is aimed at presenting the spark plasma sintering method which uses multiple electric pulses of low voltage applied in a sequence. Spark plasma sintering is considered to be one of the most advanced and promising powder consolidation methods which has been thoroughly elaborated during the past two decades. Similar investigations have been conducted at Frantsevich Institute for Problems of Materials Science since the end of the 1970s. It does not seem possible to strictly divide all electric current-assisted sintering methods into the two above mentioned groups. In some studies, consolidation was performed in two steps. The first step consisted of a short high-power electric pulse while the second one lasted longer and used electric current of lower voltages aimed at an additional sintering effect on the materials [22]. There remain several terms used to refer to the electric current-assisted sintering methods: the term “electric discharge sintering” can be sometimes used to describe a process similar to that encountered in spark plasma sintering or a process of two-step consolidation [5,22]. High-voltage consolidation is usually called electric pulse sintering [27].

### 2.1. High-Voltage Consolidation

The conducted analysis has shown that the largest number of articles on high-voltage consolidation were published by researchers from Belarus State Research and Production Powder Metallurgy Association, Minsk—Kaptsevich, Belyavin, Min’ko, Maximenko and others, who together published more than 20 articles. Research in this area was also conducted in the Moscow Engineering Physics Institute by Balankin, Gorbachev and Grigoryev, who presented more than 15 publications and developed five experimental facilities of this type.

**Figure 4:**
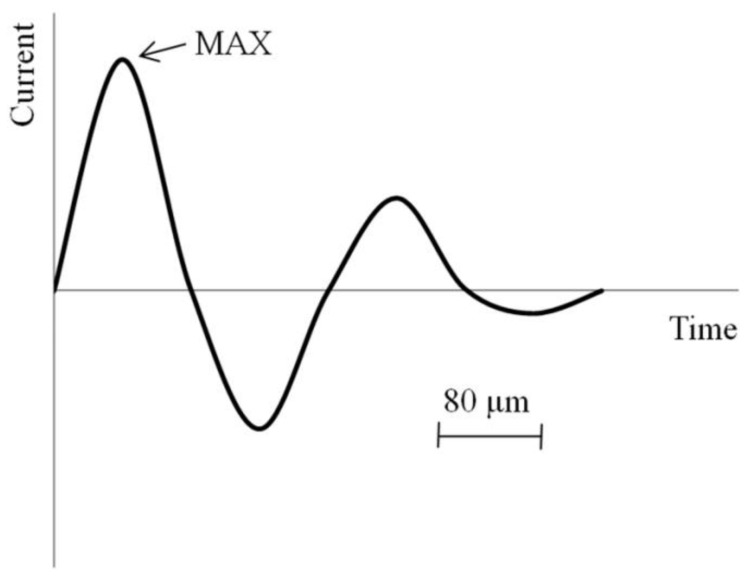
Current oscillogram in electric pulse sintering.

This section presents sintering methods, in which a short electric pulse passes through the powder to be sintered. A typical current oscillogram of the process is shown in Figure 4 [28]. Such sintering methods are referred to as electric pulse sintering. In addition, two-stage sintering is considered, in which consolidation proceeds in two steps and includes shaping by a high-voltage pulse followed by sintering in a steady-state regime for several minutes (up to 10 min).

**Figure 5:**
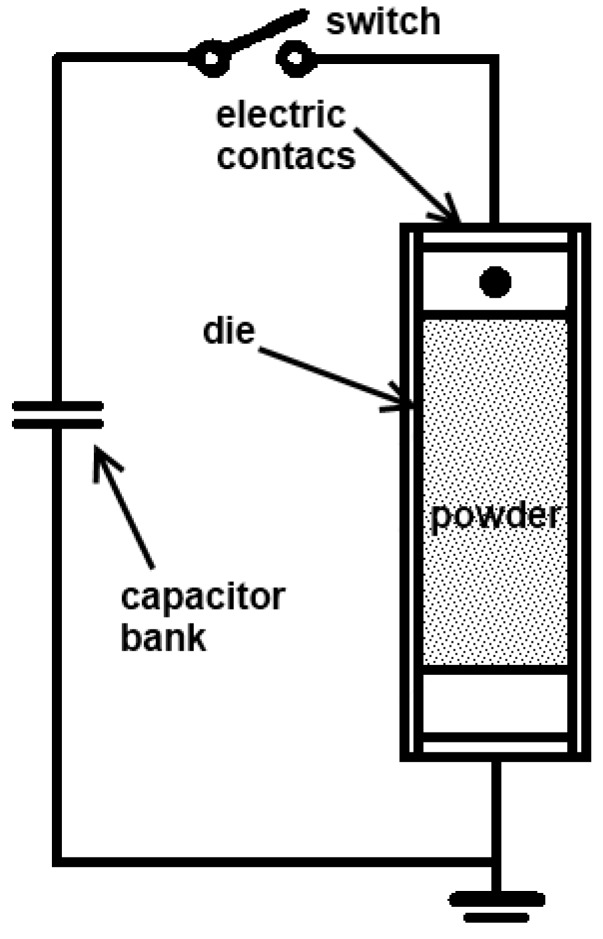
Schematic of an electric pulse sintering set-up.

At the first stage, the particle surface is cleaned making it easier for the particles to be sintered at a later stage [22]. A characteristic feature of these methods is a high voltage and a short duration of the pulse. The main advantages of this consolidation method include a possibility of grain growth retardation, which is especially important for nanosized powders, and sintering without any protective atmosphere due to short processing times. A schematic of an electric pulse sintering set-up is shown in Figure 5 [29].

**Figure 6:**
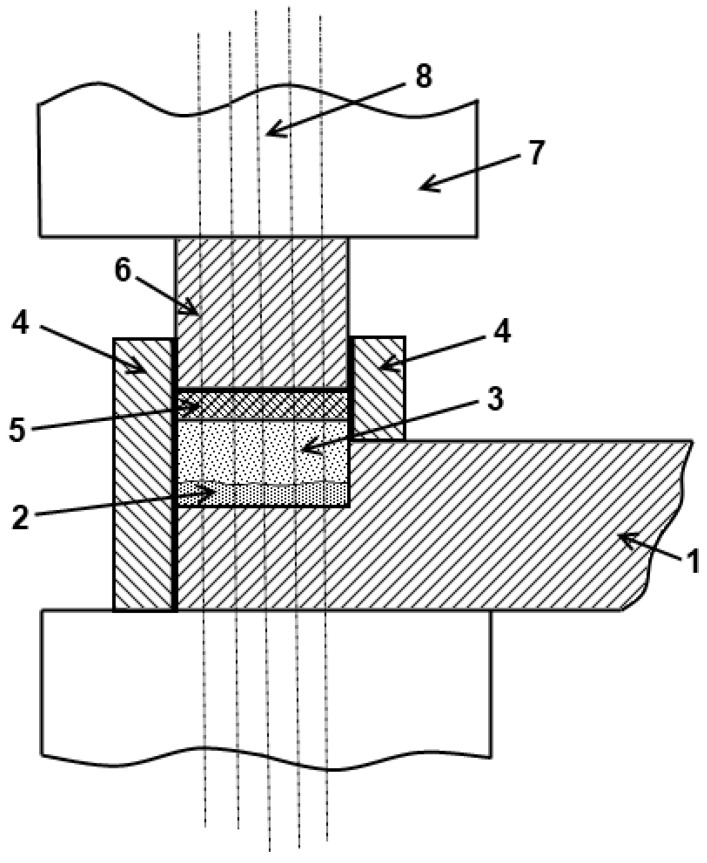
Schematic of the coating deposition set-up using electric current-assisted sintering of the powder layers: 1—tool to be coated (substrate); 2—lower powder layer; 3—upper powder layer; 4—refractory die; 5—graphite washer; 6—water-cooled snap; 7—clamps of a butt-welding apparatus or a press; and 8—the current path.

The earliest publication in USSR that described experiments, in which electric current was passing through a powder sample, is the patent of 30 November 1931 on the method of making tools with a working surface of metal carbides. The authors of that patent are Saraphanov and Liventsev [1]. The patent presents a method of making coatings from wolfram carbide powders by means of a butt-welding apparatus. In this coating deposition method, two layers are sintered and joined to the substrate, one layer is produced from a tungsten powder mixed with a metal with a lower melting temperature while the other is made of a mixture of tungsten carbide with a lower melting temperature component used in a lower content than in the first layer. A schematic of the set-up is shown in Figure 6.

**Figure 7:**
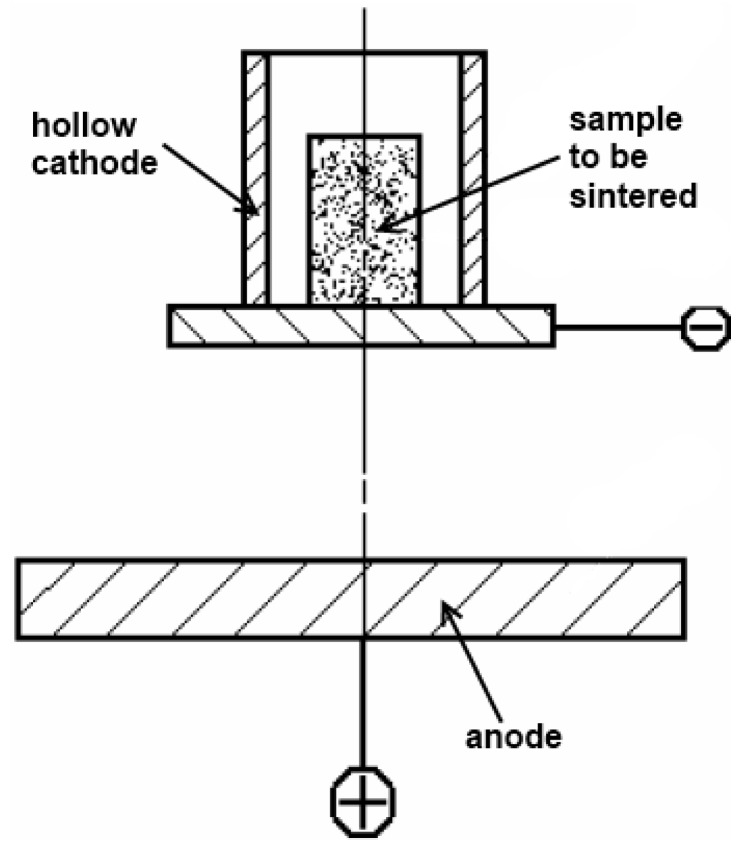
Schematic of the set-up for sintering in the plasma of a glow discharge.

An interesting electric current-assisted consolidation method was studied in Tomsk Polytechnic Institute [25]. Sintering was performed in a laboratory glow discharge set-up. The set-up is a vacuum chamber with a cathode and an anode (Figure 7). The power of a direct current source is 1 kW. The vacuum system allows adjustment of the air or ammonia pressure from atmospheric to 1 MPa. The electric current and applied pressure can be varied in this set-up. Consolidation in a glow discharge formed at certain values of residual pressure and applied potential proceeds as cations accelerated by an electric field impact on the cathode and the sample causing their heating. The sample is additionally heated by the electric current passing directly through it. The heat transferred from the cathode assembly also contributes to the total heating of the sample.

**Figure 8:**
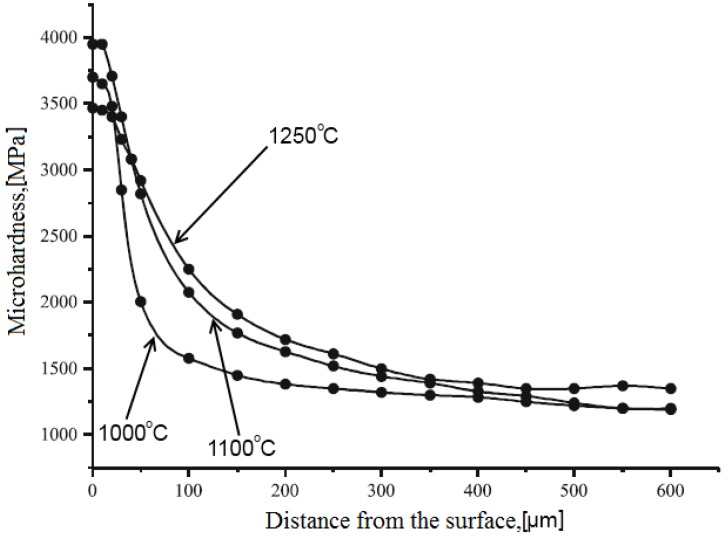
Microhardness profiles for Fe-FeTi samples sintered at different temperatures in the ammonia plasma.

Samples sintered from zirconia in a glow discharge in ammonia showed increased hardness and fracture toughness. However, when sintered in a glow discharge in air, no noticeable property improvements relative to conventionally sintered materials were observed [25]. A possibility to increase the stability of the glow discharge and prevent its transformation into an arc discharge was suggested based on the introduction of an element of variable resistance [26]. The developed sintering method was proposed by the authors as a promising technique of consolidation of antifriction materials based on Ti-alloys. The surface layer of the sample sintered in the ammonia plasma is 2.5 times harder than the rest of the sample (Figure 8).

Consolidation of powders with the help of electric current can be performed in such a manner that a layer of the powder material is sintered to a substrate and while it is rolled between two rollers, an electric pulse is passed through the assembly [12]. An earlier publication revealed the difficulties of working with ferromagnetic powders, which tended to move away from the strip influenced by an alternating magnetic field associated with the current passing through the rollers [30]. However, the problem could be solved by using a direct current.

**Figure 9:**
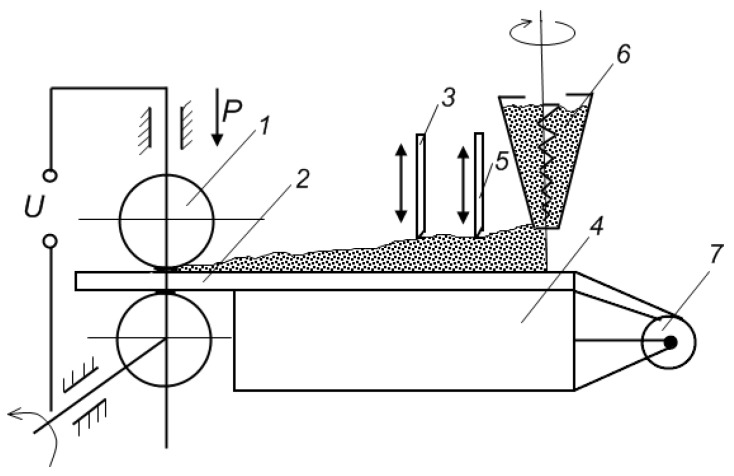
Schematic of the set-up to produce composite strips: 1—contact welding apparatus; 2—substrate strip; 3—calibrating tool; 4—dispenser box; 5—flattening tool; 6—powder feeder; and 7—pulley with a strip.

In order to study the electric current-assisted formation of coatings from powders, a series of set-ups were designed in the Institute of Powder Metallurgy, Belarus (Figure 9) [12]. The investigations showed that before the processing could be used on an industrial scale, certain challenges needed to be tackled, such as preventing powder sticking to the rollers and ensuring a better quality of the substrate surface for stronger adhesion of the coating. As possible solutions, the authors suggest using a flux compound to remove oxide films from the surface of the substrate, depositing galvanic coatings or employing specially designed rollers, e.g., graphite water-cooled rollers. As a general conclusion, the authors point to the high potential of this method and a possibility of automation.

Scientists from Belarus State Research and Production Powder Metallurgy Association, Minsk used a pilot-production facility to conduct high-voltage electric pulse sintering [31,32,33,34,35,36,37,38,39,40,41,42,43,44,45,46,47,48].

This facility had a capacitor bank [39], the pulse duration was 10–100 μs [35], current densities were in the range of 10^5^–10^6^ kA/m^2^ [38], the powder was sintered in air and in argon [40,42] after a pressing operation at 2–20 MPa [37,40]. The influence of pressure on the sintering results was studied [45]. The optimal pre-pressing parameters were found based on the measured resistivity, bending strength and radial shrinkage as functions of applied pressure.

Belyavin *et al.* [36] succeeded in consolidating long-length porous permeable parts from titanium. The dependences of the resistance of the sintered parts on the pressure and sample length were obtained by Kaptsevich *et al.* [40]. The stability of the sintered part pre-pressed at a pressure of 10 MPa is achieved when the sample height is 20–80 mm while the height to diameter ratio is in the range from 7:1 to 27:1. These results created grounds for the development of other consolidation methods for this type of sintered parts [38]. The best results were obtained when the length of the sample was increased step by step. No visible boundary was observed between different parts of the sample, which maintained uniformly distributed porosity and the same level of properties along its length.

An interesting investigation was conducted in the Joint Institute for High Temperatures of the Russian Academy of Sciences in 1999 aimed at finding the parameters of the first stage of electric pulse sintering that would make it unnecessary to perform the second stage [22]. The influence of the voltage of the properties of the sintered material was mainly studied. In another publication, Konotop *et al.* [49] considered the selection of the working parameters of pulsed current sources. The main area of the studies was sintering at voltages of 3 kV and higher. The experiments were conducted with current sources VIU-20 (Kharkov Polytechnic Institute, Kharkov, Ukraine) and VIU-50 (Kharkov Polytechnic Institute, Kharkov, Ukraine), which are used to fabricate anodes of metal-oxide-semiconductor capacitors by means of electric pulse sintering. The authors concluded that the optimal voltage range for VIU-50 was 13–15 kV, while for VIU-20 it was 30–33 kV, which is higher than the voltage normally used.

The effect of the durations of the two sintering stages on the properties of the Cu-Sn sintered material was studied by Baidenko *et al.* [50]. The results of the study show that the duration of the first sintering stage plays a decisive role. As the duration of the second stage increases from 0 to 15 s, the density of the sintered material increases from 41.5% to 97.8% (Table 1). As the duration of the second stage increases up to 20–25 s, the density remains practically unchanged. The maximum density (97.8%) is reached in the sample sintered for 30 s at the second stage. The material hardness experiences a 4-fold increase as the second stage duration increases from 0 to 15 s, the bending strength reaches maximum for the sample sintered for 15 s while the fracture toughness is maximal for the sample sintered for 25 s at the second stage.

**Table 1:** Hardness and relative density of Cu-Sn materials consolidated by two-stage electric pulse sintering using different duration of the stages.

No.	Duration of the stages, s		Relative density, %	Hardness HRB	Fracture toughness kJ/m^2^
	*t*_1_	*t*_2_			
1	10	30	93.7	82.0	5.0
2	15	30	94.6	84.0	5.4
3	20	30	94.9	85.0	5.3
4	25	30	95.0	88.0	6.1
5	30	0	41.5	20.0	1.7
6	30	5	54.8	24.0	1.8
7	30	10	74.6	55.0	2.8
8	30	15	96.6	89.0	5.6
9	30	20	96.8	92.0	6.0
10	30	25	97.1	91.0	9.5
11	30	30	97.8	93.0	5.7

**Figure 10:**
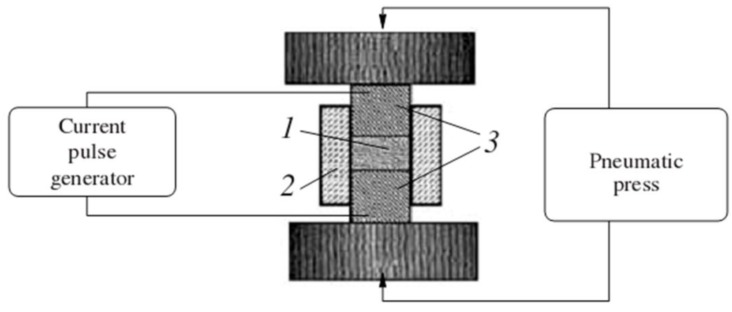
Schematic of an electric pulse sintering set-up.

Electric pulse sintering has been studied in Moscow Engineering Physics Institute since 1975 [7,8,9,10,11,51,52]. Figure 10 shows the underlying principle of the process. Powder 1 is placed in die 2 made of a dielectric material. The pressure is transferred to the sample through punches 3 also acting as electrodes. The punches carry the pulsed current over to the material being sintered in the die.

**Figure 11:**
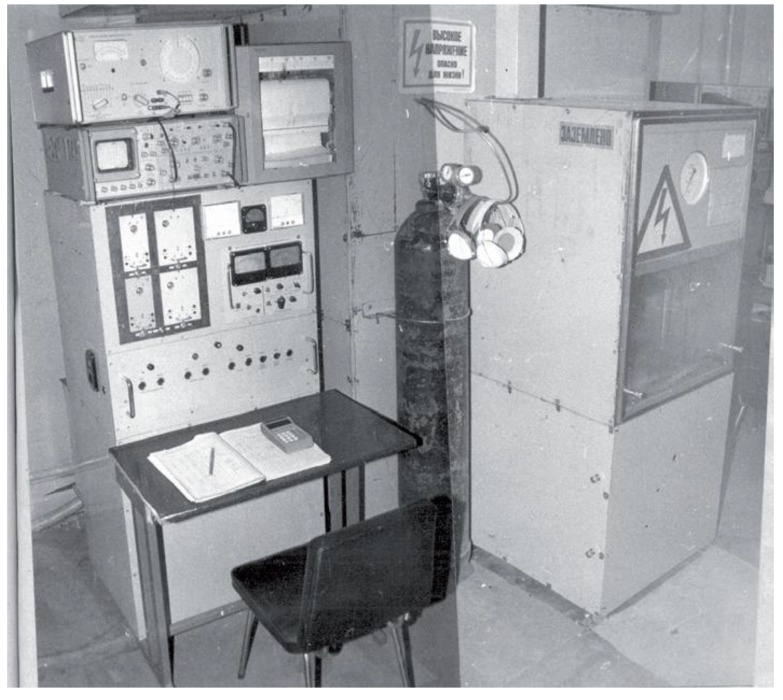
An electric pulse sintering facility.

The kinetics of electric pulse sintering was investigated by Grigoryev [28,53], who used an experimental facility shown in Figure 11.

The set-up includes an energy storage device—a capacitor bank capable of storing up to 75 kJ—a commutator (trigatron switch) capable of withstanding current pulses up to 10^6^ A, a system for temperature measurements, a device to record the densification kinetics and a pressing system (an air cylinder developing a force of 50 kN). The die is made of a ceramic non-conducting material; the punches are made of molybdenum. The sequence of processes involved in the powder sintering was studied in detail, which allowed the authors to conclude that during sintering the punches move at a constant speed. The speed increases with increasing current amplitude and applied pressure. The density of sintered material depends on the current amplitude. The process duration is 6–16 ms. A conclusion made previously was confirmed [54] on the following relationship between the discharge duration *t*_0_, densification time *t_1_* and the time necessary for the sintered sample to cool through heat dissipation *t_2_*: *t*_0_ < *t*_1_ << *t*_2_.

**Figure 12:**
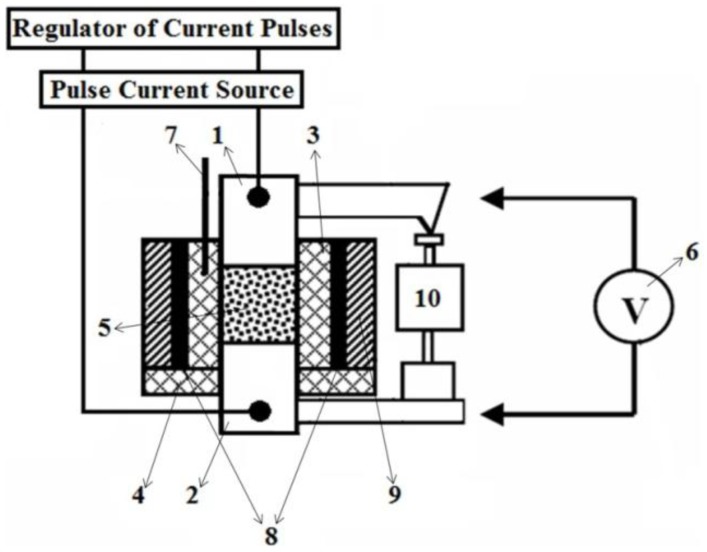
Block-diagram of the electric pulse sintering facility to sinter SiC ceramics: 1 and 2—electrodes; 3—die; 4—insulating washer; 5—powder compact; 6—voltmeter; 7—thermocouple; 8—heating element; 9—steel belt; and 10—pressure gauge.

In Dagestan State Technical University and Dagestan State University, sintering of silicon carbide ceramics was studied [55,56]. A schematic of the set-up is shown in Figure 12. The insulating dies were made of sapphire. High-power pulse generators were used as current sources.

In Frantsevich Institute for Problems of Materials Science, National Academy of Sciences of Ukraine, electric pulse sintering of powders at a constant pressure was studied [6,57]. Mixtures of iron with Mn, C, Ti, Cr and Si were investigated.

**Figure 13:**
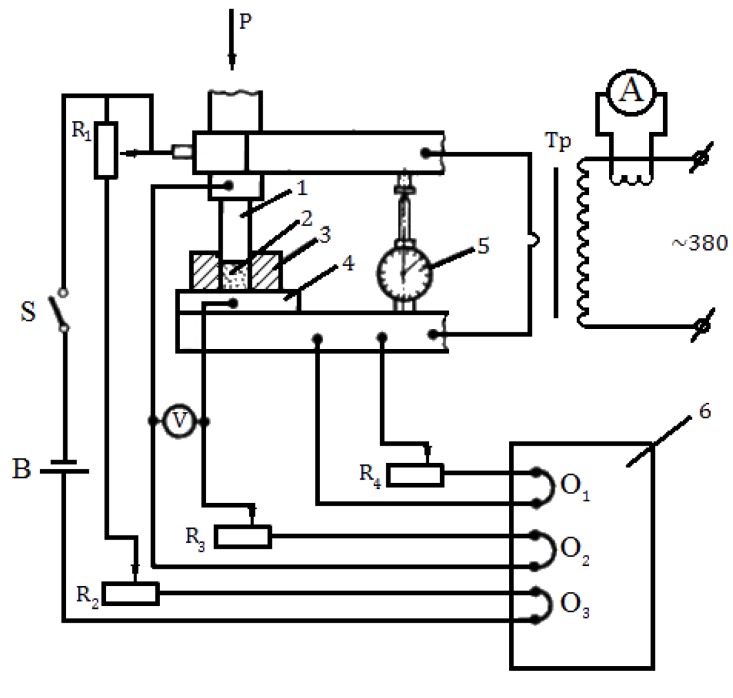
A set-up to record shrinkage during electric pulse sintering: R_1_—rheocord; R_2_, R_3_, R_4_—rheostats; V—voltmeter; Tp—transformer; A—ammeter; O_1_, O_2_, O_3_—oscilloscope probes; B—current source; S—switch; 1—movable punch; 2—powder compact; 3—die; 4—fixed punch; 5—dial indicator; and 6—oscilloscope.

The experiments were conducted using a contact welding facility of MRP-400 type. The pulsed current cycles were controlled by a circuit breaker of PISh-200 type. A schematic of the set-up is shown in Figure 13.

### 2.2. Low-Voltage Consolidation

This section reviews electric current-assisted methods that use low voltages. To refer to such methods, different terms are used, such as spark plasma sintering and electric discharge sintering. Different types of equipment and a wide range of experimental parameters have been used; many studies were conducted under an applied pressure.

A significant contribution to the development of low-voltage electric current-assisted methods was made by researchers of Frantsevich Institute for Problems of Materials Science, National Academy of Sciences of Ukraine. The majority of studies performed until 2007 in this Institute and other research organizations were conducted using a facility developed and manufactured by a research team led by Raichenko in 1976 [5]. In the following years, ERAN 2/1 set-up was also used in Frantsevich Institute for Problems of Materials Science [58,59,60,61,62,63]. In 2008–2010, Gevorkyan and Gutsalenko in Kharkov published a series of articles on sintering of ceramic materials using a hot-pressing facility with direct current heating [64,65,66,67,68]. In Nizhny Novgorod State Technical University, a team led by Mal’tsev conducted studies on sintering of powders during rolling [13,14,15,16,17,18,19,20]. In the middle of the 1960s, Frantsevich Institute for Problems of Materials Science developed a sintering method based on the use of high-frequency currents [69,70,71,72,73]. Dresvyannikov, Kolpakov, Doronin and other researchers of Kazan State Technical University conducted studies on spark plasma sintering [21,23,74,75,76,77,78,79]. Scientists from Kurdyumov Institute of Metal Physics, National Academy of Sciences of Ukraine have also made contributions to the development of this area (Andrushchik, Balakshina, Oshkaderov, Severyanina and others) [80,81,82,83,84,85,86].

**Figure 14:**
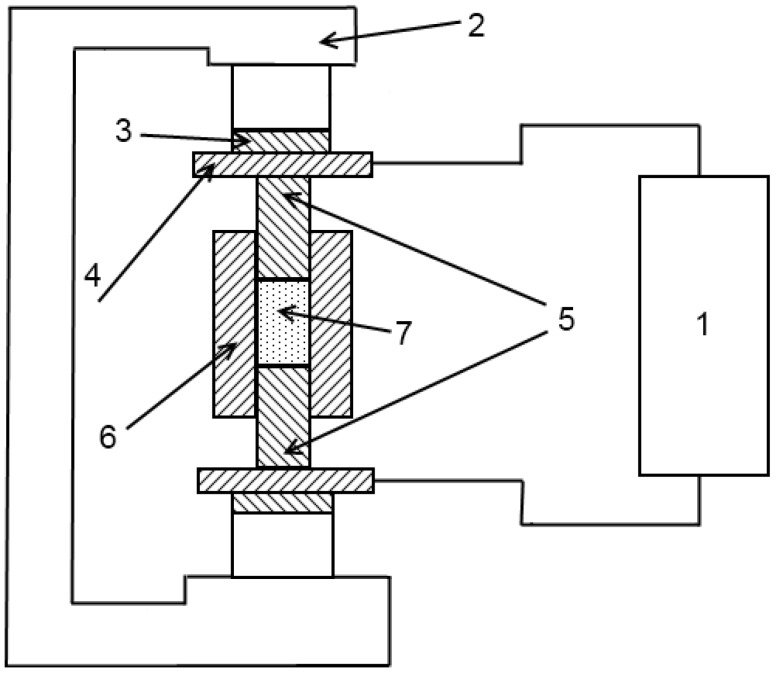
Schematic of an electric discharge sintering set-up: 1—current source; 2—press; 3—insulating plates; 4—current-carrying plates; 5—punches (electrodes); 6—die; and 7—powder compact.

In 1976, sintering of binary powder mixtures was studied in Frantsevich Institute for Problems of Materials Science, Ukraine [5]. A facility developed in this Institute to perform the sintering experiments is shown in Figure 14.

The studies were aimed at comparing the defect state of the materials obtained under different conditions. In the first series of experiments, the current density was varied from 397 to 624 A/cm^2^ while other parameters remained constant (frequency of 2.6 kHz, sintering time of 30 s, applied pressure of 85–88 kg/cm^2^). In the second series, the sintering time was varied from 10 to 45 s while the current density was constant (624 A/cm^2^).

The same year Raichenko and his colleagues studied electric discharge sintering of aluminum, copper, iron [87] and electrolytic nickel [88], and a year later, in 1977, reactive sintering of the Cu-Al powder mixture by the electric discharge method was reported [89]. Sintering was performed using the set-up of Figure 14.

Raichenko *et al.* [90,91,92], Svechkov *et al.* [93], Baidenko *et al.* [94,95,96] and Ryabinina *et al.* [97,98] conducted a large number of experiments using this set-up.

Raichenko and his colleagues [99,100] studied the processes occurring at the inter-particle contacts during electric discharge sintering in the case of spherical particles. They also sintered tool materials by electric discharge sintering and studied the influence of the content of an abrasive component on the mechanical properties and microstructure [101].

Ryabinina (Orenburg Polytechnic University) studied the interaction of the punches (electrodes) with a metallic powder [102,103,104,105] and sintering of composite materials [106,107,108]. The electric discharge sintering experiments were conducted using the facility designed in Frantsevich Institute for Problems of Materials Science, Ukraine.

Scientists from Kurdyumov Institute of Metal Physics, National Academy of Sciences of Ukraine (Andrushchik *et al* [80,81,82]) investigated the governing relationships of electric contact sintering of pre-pressed powder compacts for an extended period of time.

**Figure 15:**
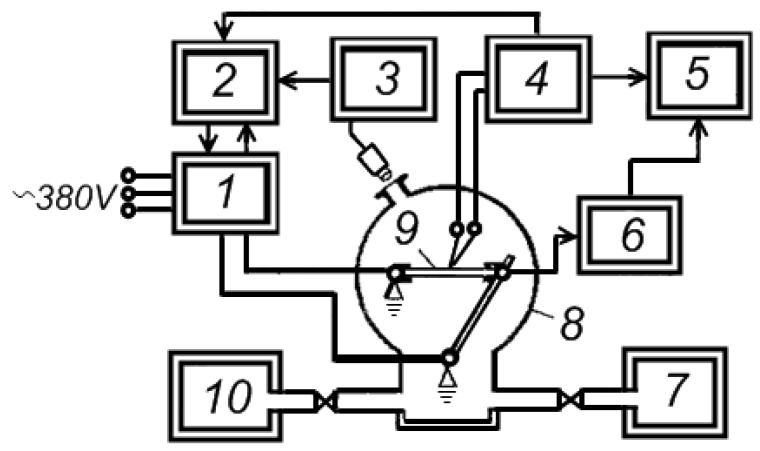
Principle scheme of the electric contact sintering set-up: 1—power unit; 2—control unit; 3—photoelectric pyrometer; 4—temperature recording unit; 5—recording potentiometer; 6—electrical dilatometer; 7—gas system; 8—working chamber; 9—sample; and 10—vacuum station.

These investigations allowed the development an automated prototype set-up to sinter ring-shaped samples (Figure 15). Sintering was performed in hydrogen.

In 1985, Hermel *et al* [83], Andrushchik *et al* [84,85] conducted research on electric contact sintering of iron powders. The method uses electric current (alternating or direct) that passes directly through the sample, which can be 50–150 mm long and can have a cross-section of 50 mm^2^. By varying the pulse duration, the authors changed the power input in the sample, which in turn, allowed control of the heating rate. The heating rate could be varied between 10 and 500 °C/s, the voltage between 0 and 50 V; the maximum current was 1500 A. The experimental set-up consists of three main parts: a power unit, a control and automation unit and a heating chamber (Figure 15) [86].

The same set-up was used by Andrushchik and Oshkaderov [86] in 2003 to study electric contact sintering of iron-based alloys.

**Figure 16:**
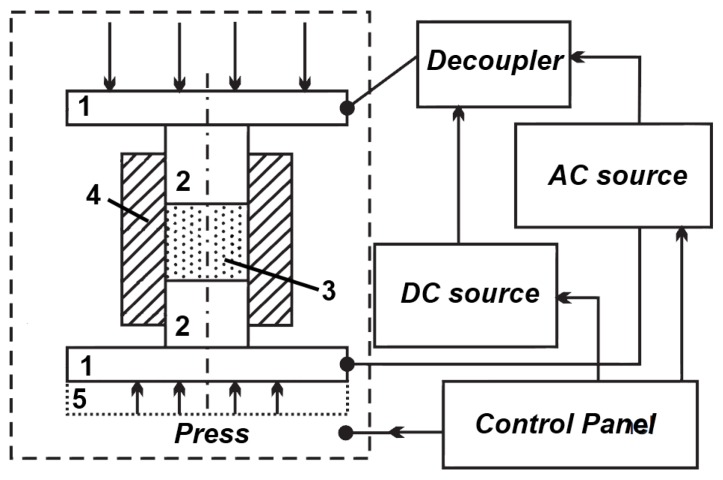
ERAN 2/1 installation for electric discharge sintering: 1—current-carrying plates of the hydraulic press; 2—punches (electrodes), graphite MPG-6; 3—powder mixture; 4—die, MPG-6 graphite; and 5—press.

Electric discharge sintering was also conducted using ERAN 2/1 facility of Frantsevich Institute for Problems of Materials Science, National Academy of Sciences of Ukraine [58,59,60,61] shown in Figure 16. This facility allows heating samples 8 mm in diameter up to 1700 °C in 90–120 s.

The samples were sintered under the following conditions: an alternating current of 0.3 kA, a direct current of 1.1 kA and a pressure of 80 MPa were used. Electric current was applied to the powder compact using graphite tooling. When graphite tooling is heated up to 1100 °C, it can react with oxygen to form CO, which serves as protective atmosphere for the consolidated samples.

This set-up was subsequently used to sinter different materials. In Frantsevich Institute for Problems of Materials Science, National Academy of Sciences of Ukraine, titania ceramics was sintered using this method. In particular, Petukhov [62] studied the influence of the processing parameters on the sintering behavior of the TiN-TiB_2_ composites. Sintering was performed in air in a graphite die applying direct and alternating currents simultaneously. The density of the direct current was 5 × 10^6^ A/m^2^ while that of the alternating current was in the (2.0–2.5)·10^6^ A/m^2^ range, the frequency was 5 kHz, the heating rate was 28–42 °C/s and the total sintering time was 180 s. The applied pressure was 60–80 MPa. This helped establish an optimal electric contact between the graphite punch and the powder particles. The same material was also sintered using a sintering facility made by Sumitomo (Japan). In these experiments, a pulsed current was passed through the sample placed in a graphite die. The powders were treated in vacuum of 0.133 Pa to remove hydrogen. The total sintering time was 480–670 s, the heating rate was 2–4 °C/s and the applied pressure was 40 MPa. The first sintering method was shown to be more efficient resulting in a more uniform microstructure of the sintered samples of varied density and better mechanical properties.

A TiN powder was sintered using ERAN 2/1 set-up [63] at a heating rate of 425 °C/min and a maximum temperature of 1500 °C. No isothermal holding of the sample at the maximum temperature was used.

In 2009, a collaborative work was done by several research organizations in Russia (Nizhny Novgorod State Technical University, Russian Federal Nuclear Center—The All-Russian Research Institute of Experimental Physics and Institute of Metallurgy, Russian Academy of Sciences) on sintering of tungsten pseudoalloys [109,110]. Consolidation was performed by spark plasma sintering using a Dr. Sinter SPS-625 (SYNTEX Inc, Kawasaki, Japan) and by conventional pressure-less sintering. In both cases, before sintering the powders were hydrostatically pressed at a pressure of 50 MPa.

Low-voltage sintering was studied in Kazan State Technical University by Dresvyannikov [74], Kolpakov *et al.* [75,76]. They synthesized Fe_3_Al by spark plasma sintering using a SPS-511S apparatus (SYNTEX Inc, Kawasaki, Japan) [74,75], sintered Al_2_O_3_-Fe cermets [76] and synthesized different ceramic materials [23]. The applied pressure in all experiments was 50 MPa. The heating time was 12 min. The samples were held at the maximum temperature (1000 °C) for 5 min.

Dresvyannikov *et al.* [77], Kolpakov *et al.* [21] sintered Fe-, Co- and Al-based precursors using a spark plasma sintering facility manufactured by Thermal Technology LLC (Santa Rosa, CA, USA). The samples were held for 30 min in a graphite die at a pressure of 60 MPa and at a temperature of 1200 °C. The heating rate was 50 °C/min and the residual pressure in the sintering chamber 0.8 × 10^−2^–3 × 10^−2^ Hg.

Petrova *et al.* [78,79] investigated sintering of oxide nanopowders at different pressures and temperatures using the same facility.

Sintering of metal-ceramic materials by high-frequency currents (70 kHz) was studied in 1965–1966, in particular, a LZ-67 high-frequency facility (Leningrad factory of high-frequency systems, Sankt-Petersburg, Russia) was employed to sinter the piston-rings of the ZIL-158 motor [69,70].

For sintering of ring-shaped parts, a rotary set-up was specially constructed including a multi-turn inductor (Research and Production Association of Automotive Industry Autoprom). Sintering was conducted under an applied pressure.

Nikitina conducted sintering at the following parameters: *I*_circuit_ = 0.7 A; *I*_anode_ = 1 A; *U* = 1 V [106]. The samples were heated at a rate of 20 °C/min until indication of melting appeared. No holding at the maximum temperature was used; the samples were cooled in air. During sintering, the electric parameters changed and their final values were as follows: *I*_circuit_ = 0.6 A; *I*_anod*e*_ = 1.2 A; *U* = 0.8 V. The optimal heating regime for the Fe (balance)-1.35%, C-2%, Cu-4% composition corresponded to a combination of the following conditions: *I*_circuit_ = 0.2 A; *I*_anode_ = 0.9 A; *U* = 7 V and a holding time of 38 s [107].

In 1989, Plekhanov [71] studied the process of making sealing rings on the basis of iron using sintering by high-frequency currents.

Sintering of pre-pressed rings was performed through 3-step heating, which included indirect heating, heating in a transverse magnetic field by an inductor and heating by a ring inductor. The author developed and assembled a set-up for sintering ring-shaped samples by high-frequency currents.

Ermakov and Krautman [72] studied the sintering processes induced by high-frequency currents in steel powders.

In Orenburg State University, peculiarities of additional densification were considered for the compacts sintered form iron powders by high-frequency currents [73].

**Figure 17:**
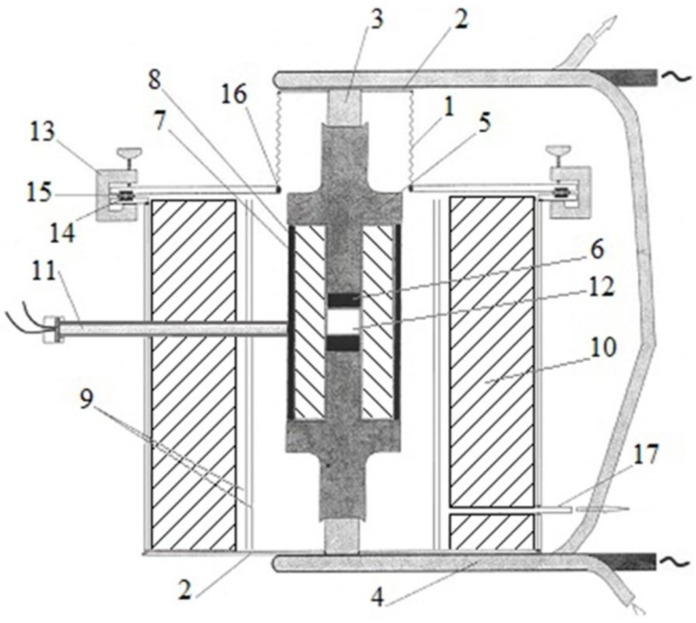
Schematic of the hot-pressing facility using direct passage of alternating current of industrial frequency through the powder to be consolidated manufactured by Research and Development Enterprise “Cermet-U” (Kharkov, Ukraine): 1—sylphon (steel Cr_8_Ni_10_Ti); 2—flanges (steel Cr_18_Ni_10_Ti); 3—self-cooled electrodes (copper alloy); 4—water-cooled electrodes (copper tube); 5—punches (graphite); 6—spacer (graphite), 7—split die (graphite); 8—bushing (a carbon-based composite); 9—shields (Mo-alloyed steel); 10—thermal insulation; 11—thermocouple W-Re–5/20; 12—sample to be consolidated; 13—clamps (dielectric); 14—gasket (rubber); 15—gasket (dielectric); 16—vacuum welds; and 17—inlet (for a vacuum pump).

In Kharkov, Gevorkyan and Gutsalenko [64,65,66] investigated the sintering mechanisms of ceramic powder materials during hot-pressing, in which electric current was allowed to pass through the powder (Figure 17). The powders were heated at a rate of 50, 250 and 500 °C/min up to 1400 °C without any binders [106]. The samples were 19 mm in diameter and 5 mm high. They were held at the maximum temperature for 2 min.

**Figure 18:**
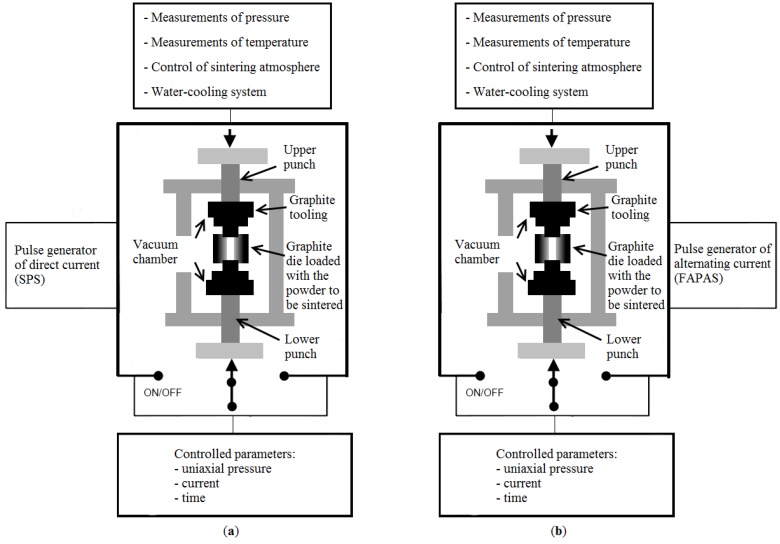
(**a**) Schematic of spark plasma sintering (SPS) and (**b**) field activated pressure assisted synthesis (FAPAS) facilities.

**Figure 19:**
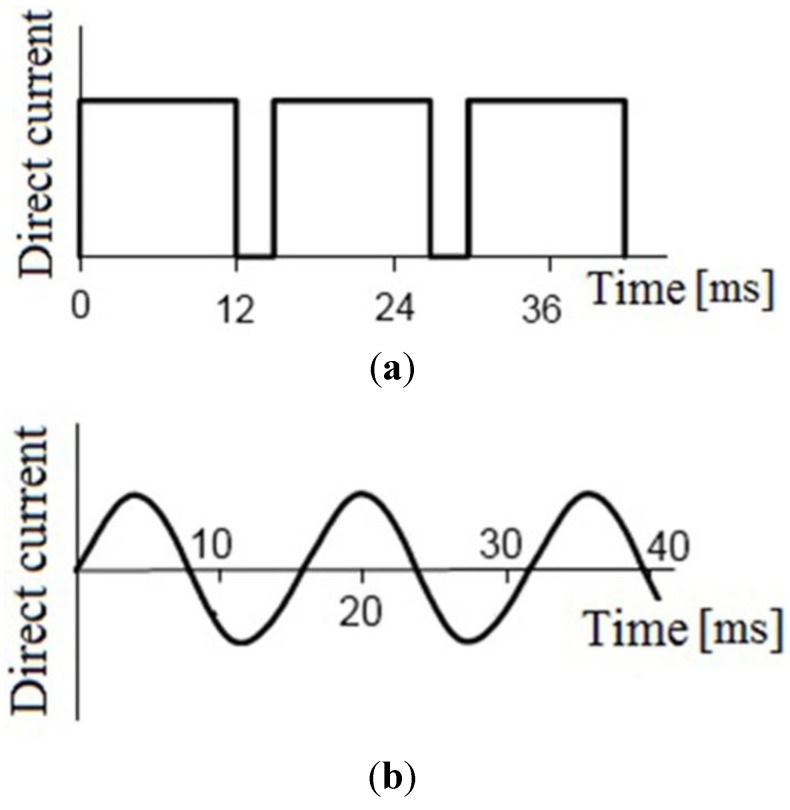
(**a**) SPS mode: 100 kN, 15 V, 5 kA, direct current, pulse period 14 ms, relative pulse duration 12:2; and (**b**) FAPAS mode: 100 kN, 10 B, 1.75 kA, alternating current 60 Hz. (Dijon-Belfort, France; Davis, CA, USA).

In another publication, Gevorkyan and Gutsalenko [67] considered theoretical aspects of electroconsolidation by electric current directly passing through submicron and nanopowders under applied pressure. Different sintering techniques were compared, in particular, the FAST (SPS) technique and FAPAS (Field Activated Pressure Assisted Synthesis) that uses alternating current of industrial frequency (Figure 18 and Figure 19).

**Figure 20:**
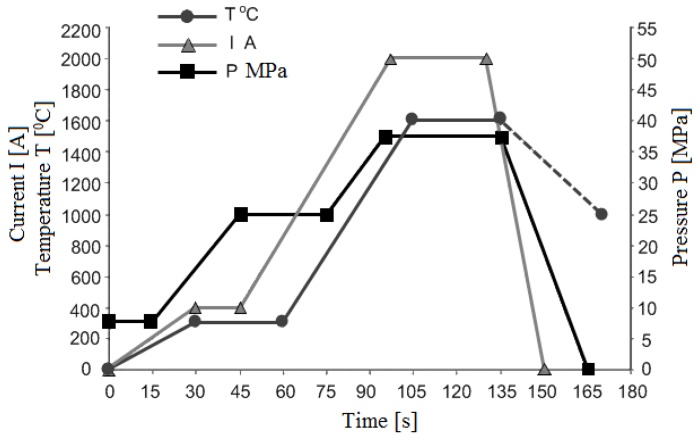
Hot-pressing conditions of Al_2_O_3_-WC nanopowder mixtures (50/50 wt %).

In 2010, Gevorkyan and Gutsalenko developed a consolidation method for refractory nanopowders based on the use of electric current during hot-pressing. Experiments were conducted using a composite Al_2_O_3_-WC mixture at a voltage ranging from 5 to 10 V and at an alternating current of 1500–2000 A [24,68]. Consolidation resulted in the formation of compacts of uniform microstructure and density due to the effect of electric current passing directly through the sample. Figure 20 shows the variations in the sintering parameters during the process.

**Figure 21:**
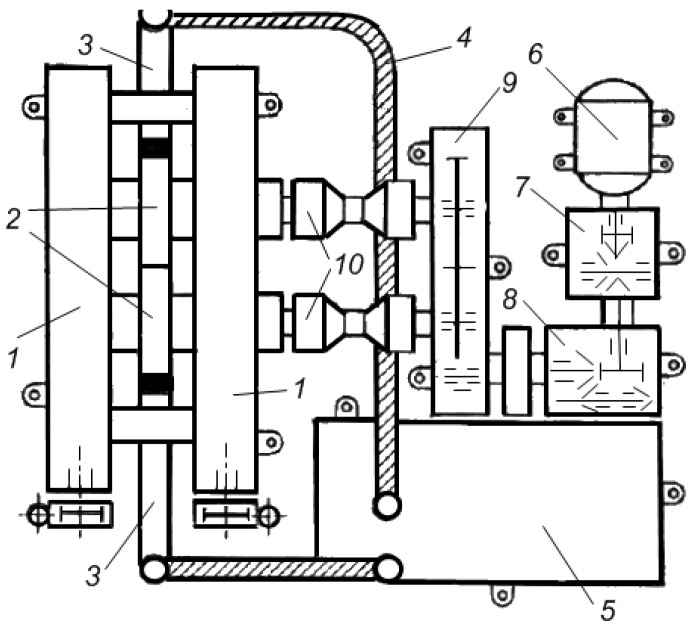
Schematic of the set-up for electric pulse sintering of powders during rolling.

In Nizhny Novgorod State Technical University, Mal’tsev and his group studied electric current-assisted sintering of conducting powders during powder rolling [13,14,15,16,17,18]. A set-up was developed and manufactured (Figure 21) [19,20,111]. This set-up allows conducting sintering of the powders by electric current simultaneously shaping them by the rollers. The heat dissipation is balanced by high heating rates, with which it is possible to increase the voltage amplitude of the current pulse between the powder and the rollers (electrodes).

The set-up includes a rolling mill with a working stand 1, rollers 2, contact elements 3, electrodes 4, an energy block 5 and a measuring unit. The rolling mill has an asynchronous motor 6 of a power of 12 kW with a start and reverse device, a step-down spiral gear 7, a four-step gear-box 8, a transfer gear-box 9 with cardan rods 10, which are connected with the rollers through insulating spacers (Figure 21).

**Figure 22:**
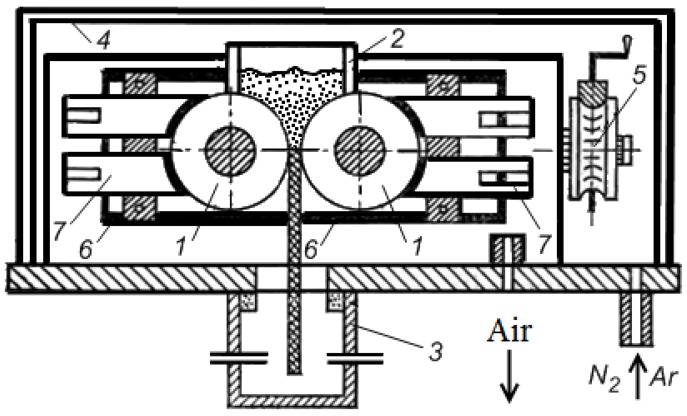
Working stand.

A schematic of the working stand is shown in Figure 22. The working stand can support horizontal rollers 1 (electrodes) 0.1 or 0.2 m in diameter made of hardened steel CrWMn or 35CrMnSiN. Rollers with demountable working barrels made of a conducting material as well as a bin for the gravity feed of the powder 2 can be installed. A hollow leakproof cylinder 3 is included to cool the strips. The working stand can be placed in a gas chamber 4 to create the required atmosphere (argon, nitrogen). Inert gases are used to purge the system, vacuum pumping (by forevacuum pumps) is simultaneously used. In order to control the thickness of the strip, the working stand contains pressure screw devices 5. These devices are placed opposite to each of the roller necks. The working stand is insulated by special spacers 6 from the rollers and contact elements 7. The contact element is a sliding plate and is made of MG-2 or a Mo-Cu pseudoalloy.

The authors have shown that by changing the parameters of the pulsed current, the power of the set-up can be varied and, therefore, the heating of the powder can be controlled. The voltage in the secondary circuit was varied in the interval of 3.15–20.9 V, the maximum effective current was 3.82 kA, the pulse duration was 0.02–0.26 s and the pauses between the pulses were from 0.02 to 0.40 s.

**Figure 23:**
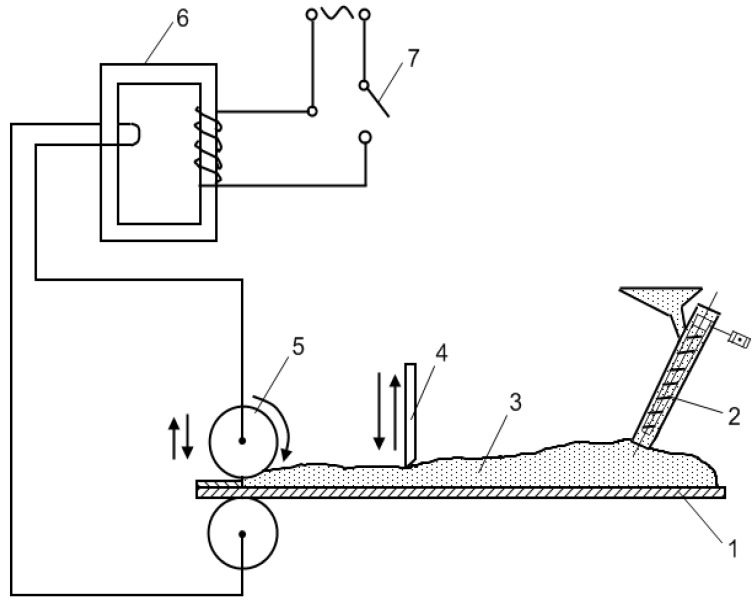
Schematic of the set-up for powder rolling: 1—table; 2—conveyer worm; 3—powder; 4—powder adjuster; 5—rollers (electrodes); 6—transformer; and 7—switch.

An earlier publication on electric current-assisted sintering of conducting powers during rolling was co-authored by Semenov, Semenov and Kondratov [112]. In 1964, in Zaporozhie, a facility for depositing antifriction, sealing and protective coatings on metallic parts was developed (Figure 23). In this work, powders of copper with additions of Pb, Sn, Fe and graphite were investigated.

The samples were sintered by applying a force of 6.58 kN between the rollers and a current of 8000–12000 A. Pulses 0.38 s long were applied every 0.02 s. The table moved at a velocity of 20 mm/s, the voltage was 380 V.

Frantsevich Institute for Problems of Materials Science, National Academy of Sciences of Ukraine in collaboration with National Institute for Materials Science, Japan investigated SPS of the TiN-TiB_2_ composites using a Dr. Sinter 1050 facility (Sumitomo Coal Mining Co., Tokyo, Japan) [113]. This facility allows recording electric current, voltage, temperature and residual pressure in the chamber in real time during sintering.

Experiments were conducted in vacuum (7.5 × 10^−2^ torr) under a pulsed current (the ratio of the ON:OFF durations was 12:2). The samples were heated up to 900 °C at an applied pressure of 60 MPa, which was further increased up to 80 MPa. The heating rate was 140 °C/min from room temperature up to 700 °C, during further heating up to 1600 °C, higher heating rates were used (112.5–300 °C/min). The sample was held at the maximum temperature for 3 min. The total heating cycle was 12–17 min.

**Figure 24:**
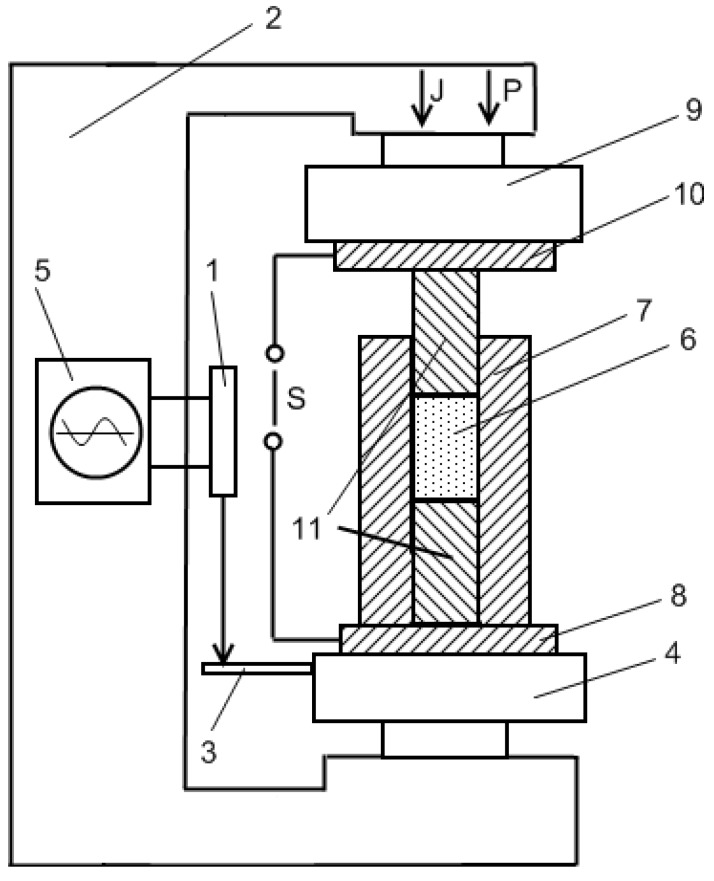
Schematic of an electric discharge sintering set-up.

Another investigation of the influence of pressure and current density on densification during electric discharge sintering was conducted in Frantsevich Institute for Problems of Materials Science, National Academy of Sciences of Ukraine using a facility shown in Figure 24 [114]. An electric measuring element 1 is fixed on the frame of the set-up 2 and on a solid metallic strip 3 connected to the slider 4. A change in density during sintering is registered by an oscillograph 5. A powder 6 is poured into a die 7, which is placed on a conductive plate 8. The pressure in the die was created by a plunger 9, which was connected to a conductive plate 10. Punches 11 conduct electric current, which is a combination of direct and alternating currents, to the powder compact.

**Figure 25:**
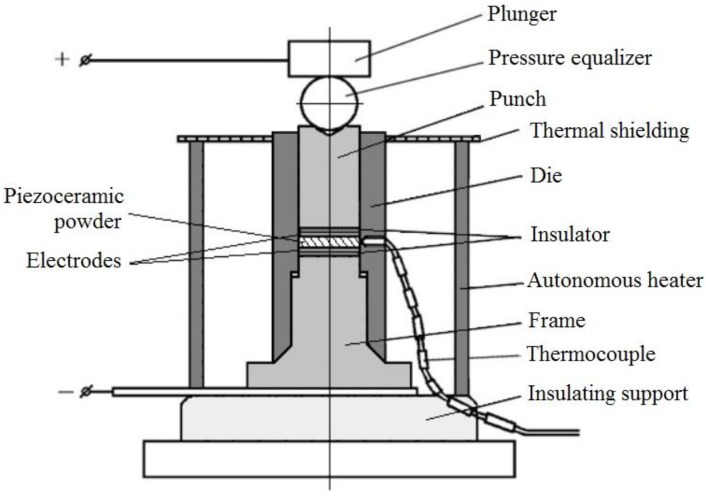
A set-up for pressing, sintering and polarization of piezoceramic materials: 1—plunger; 2—pressure equalizer; 3—punch; 4—thermal shielding; 5—die; 6—insulator; 7—a piezoceramic powder; 8—electrodes; 9—autonomous heater; 10—frame; 11—thermocouple; and 12—insulating support.

Ekimov, Amelchenko, Fillipov and Utenkov studied the piezoelectric materials [115]. A scheme of the experimental set-up that was used in their experiments is shown in Figure 25.

Stepanova *et al.* [116], Lyakhov *et al.* [117], Dudina *et al.* [118] conducted studies on sintering of composite powders in an SPS apparatus (Sumitomo Coal Mining Co., Ltd, Tokyo, Japan).

In 1988, Belousov, Pilipchenko and other researchers of Ivano-Frankivsk National Technical University of Oil and Gas studied the self-propagating high-temperature synthesis in Ti and C mixtures mixed in a 4:1 ratio [119]. The set-up used in their experiments was a vacuum chamber containing a quartz glass die and two graphite punches. During the experiments, the voltage, current, applied pressure and temperature of the powder sample were monitored. The voltage was in the range of 2–24 V.

**Figure 26:**
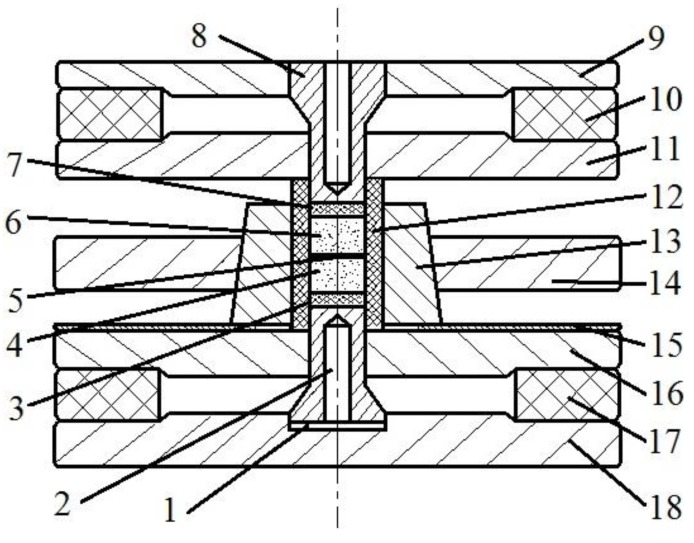
Technological assembly for sintering of diamond-containing composite materials. Design details: 1—copper spacer; 2—lower punch; 3—lower thermally-insulating plug; 4—lower briquette; 5—separating gasket; 6—upper briquette; 7—upper thermally-insulating plug; 8—upper punch; 9—upper stake; 10—upper rubber gasket; 11—upper die; 12—lithographic stone container; 13—split die; 14—fastening case; 15—insulator; 16—lower die; 17—lower rubber gasket; and 18—supporting block.

In Bakul Institute for Superhard Materials, a set-up was developed for sintering of diamond-containing composite materials (Figure 26) [120]. In this set-up, the sample, which has been previously compressed in a container made of lithographic stone, experiences the direct action of electric current passing through it. This method is suitable for consolidating materials at pressure up to 0.5 GPa very rapidly (in 10 s), which allows the mechanical properties of the diamond phase to be retained, usually susceptible to conditions of hot-pressing. The proposed method is relatively cheap and simple presenting an alternative to the current technology. However, sintering of large-scale parts is difficult due to high temperature gradients.

**Figure 27:**
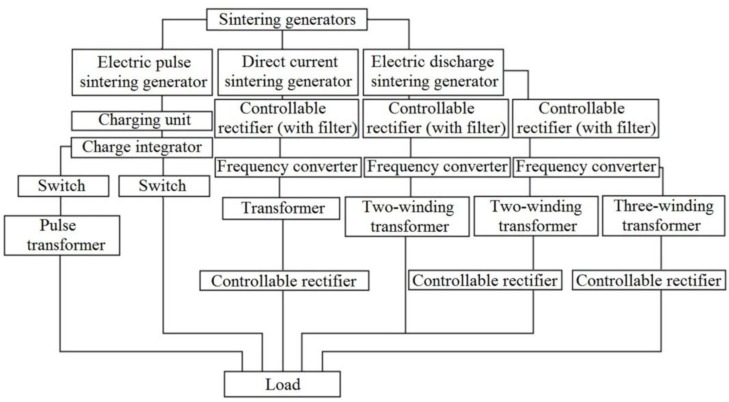
Classification of generator circuits used in sintering facilities.

Kurashko *et al.* [121] of the Institute of Pulse Processes and Technologies, National Academy of Sciences of Ukraine analyzed the generator schemes used for sintering, searching for a more efficient solution. The classification of the generators is presented in Figure 27. The generator used in electric pulse sintering has an electric diagram showing how the load is connected in the circuit. A capacitor is connected though a switch to the load and is charged up to a selected voltage. A series of decaying pulses pass through the load.

**Figure 28:**
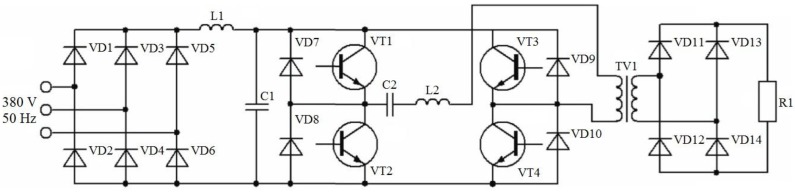
Electric diagram of a generator used for sintering experiments. The generator has a transistor-based bridge inverter.

In this paper, an electric diagram of a generator used for low-voltage sintering experiments is given (Figure 28). The generator has a transistor-based bridge inverter. This scheme allows for a flexible control of the ratio of the direct and alternating current components passing through the sample. A generator with a power of <10 kW can be developed.

It can be concluded that the studies in the area of electric current-assisted sintering of powder materials using electric currents directly passing through the sample were actively developing throughout the whole period considered. Several sintering set-ups were designed; some of them were modernized and were used until recently. Modern low-voltage sintering facilities and sintering parameters were described. A large amount of work was done to optimize the sintering conditions for different material systems. Several authors performed a comparative analysis of the sintering results produced on one and the same material consolidated by different sintering techniques.

## 3. Materials Processed by Electric Current-Assisted Consolidation

### 3.1. Processed Metals and Alloys

Electric current passing through an assembly of powder particles causes intensive mass transport in the zones of inter-particle contacts. The mass transport is accelerated due to possible melting and vaporization of the metal in the contact zones. As a result, rapid sintering of a single-phase powder occurs; in multi-component systems, conditions for alloying and chemical reactions (reactive sintering) are created [29]. Liquid-phase sintering of multi-component systems can be carried out; in this case, the sintering temperature should be higher than the melting temperature of the component with a lower melting temperature while it should be lower than the melting temperature of the refractory component of the mixture. If oxidation should be avoided, sintering needs to be carried out in vacuum or an inert or reducing atmosphere. In metals consolidated by electric current-assisted methods, better mechanical properties can be achieved compared to those sintered by other techniques.

#### 3.1.1. Fe Powders Processed by Electric Current-Assisted Consolidation

Powder materials based on iron are promising for the development of antifriction materials and high-strength alloys. The main challenge in the preparation of these materials is to provide sufficient wear resistance and improve other physical and mechanical properties [26].

In order to produce steels and alloys containing a high concentrations of iron, optimal sintering parameters should be found for Fe powders. Electric discharge sintering of iron powders was carried out and the influence of the temperature, holding time and applied pressure on the microstructure and mechanical properties of porous iron was studied [84]. It was found that after electric current-assisted consolidation, porosity in iron decreases to a lesser extent than in conventionally annealed samples at a constant temperature. However, the pores in the compacts sintered by electric current are more uniformly distributed. Increasing the duration of isothermal annealing to more than 110 s does not significantly alter the properties of the sintered material. A sintering temperature of 1100 °C and a short holding time at a constant temperature are insufficient to form a microstructure providing the required mechanical properties. The total number of pores and the pore shape coefficient are higher for the sample sintered at this temperature than those for the sample sintered at 1300 °C. For this reason, sintering at 1300 °C allows the achievement of better mechanical properties.

Electric discharge sintering of iron powders and an iron-based alloy containing 2.8% C; 7.84% Mn; 28.65% Cr; 1.9% Ti; 0.47% Si was studied [6]. A pre-pressed sample having density of ρ = 3.24 g/cm^3^ was sintered in a contact welding apparatus of MRP-400 type (“Zavod Elektrik”, Sankt-Petersburg, Russia). The pulse duration was 0.1 s and the interval between the pulses was 0.1 s. The maximum density of the compact was reached at a pressure of 26 MPa, a current of 15.5 kA, a voltage of 6.4 V, a total time, which includes cold pressing, the sintering cycle and subsequent holding, of 7.4 s, and a cycle of 4.5 s (the total duration of pulses and intervals between them). A dramatic increase in the sample density is observed at 670–720 °C. The material sintered under the described conditions possessed a non-equilibrium structure, high hardness, wear resistance and high density of 7.55 g/cm^3^. An existing possibility of increasing the density of the sintered material was confirmed by raising the applied pressure or the density of current passing through the sample [6].

Atomized powders of low-alloyed steel are difficult to press into compacts. This is due to the particle shape and high hardness. Sintering of an iron-based alloy containing 0.04% Cr, 0.63% Mn, 0.57% Mo, 2.7% Ni, 0.45% C was presented [122]. The processing, microstructure and properties of ring-shaped samples consolidated by the electric current-assisted method were also described [86]. Two iron-based powders were studied, one containing 0.05% Cr, 0.29% Mn, 0.53% Mo, 1.92% Ni and the other containing 0.32% Cr, 0.52% Mn, 0.3% Mo, 0.25% Ni. The granules were 0.16 mm in diameter; a coarser fraction (10%) with a diameter of 0.4 mm was added. The powders were pressed at a pressure of 400–800 MPa upon addition of 0.2%–0.5% of graphite in an atmosphere of dehumidified hydrogen. It was found that densification occurs during heating at a high rate and the duration of the isothermal dwell has no noticeable influence on the density. The minimum porosity was observed in the sample pressed at a pressure of 800 MPa. Samples of different compositions sintered at 1473 K and 600 MPa show spherical pores, smaller pores being observed in the alloy of composition (1). This shape of the pores indicates the presence of a liquid phase during sintering. Carbon present in the alloy increases the values of microstrain from 10^−5^–10^−4^ to 10^−3^, which can be explained by changes in the microstructure and phase composition of the material, such as the formation of graphite inclusions [86].

Methods of powder metallurgy are used to manufacture antifriction parts for assemblies, in which a lubricant is constantly necessary. An alloy containing 95% of iron and 5% of titanium was sintered in the plasma of a glow discharge [26]. The samples were double-sided pressed at a pressure of 600 MPa. After pressing, they were sintered in the air or ammonia plasma at 1000–1250 °C, electric current and voltage ranging from 0.1 to 0.6 A and from 450 to 650 V, respectively. The residual pressure was 0.1 Pa during sintering. The sample was held at the maximum temperature for a certain time. Titanium is more efficiently introduced into the powder mixture when ferrotitanium is used. The alloy sintered from Fe-FeTi has a more uniform porosity than the alloy sintered from Fe-Ti. The surface of the sample sintered in the ammonia plasma is 2.5 times harder than its interiors. This hardened layer is 100 µm thick and is composed of nitrides.

#### 3.1.2. Cr-Based Systems Processed by Electric Current-Assisted Consolidation

Chromium is a component of alloys and a surface-alloying element of steels. When a Cr powder is sintered to the surface, a Cr-rich layer is formed possessing higher hardness and corrosion resistance compared to the interior of the steel part. Parts of pure chromium are not manufactured because of its brittleness. However, it is important to study its sintering behavior.

Electric discharge sintering of chromium of PCh1M grade (25 µm) was studied [81]. The heating with a rate of ~10^3^ K∙s^−1^ was due to direct passage of current through the sample and was followed by an isothermal dwell for 1.8 × 10^3^ s at 1400 °C. A lower sintering temperature of 1300 °C and a shorter sintering time were also used [82]. During the initial sintering stage, the porosity increased (during the first 60 s), then started to decrease. The dislocation density decreased by two orders of magnitude compared to the chromium powder. As the concentration of dislocations in the walls decreased and that of random dislocations increased, a homogeneous low-porosity structure formed.

#### 3.1.3. Ni-Based Systems Processed by Electric Current-Assisted Consolidation

Cu-Ni powder mixtures are used to fabricate metallic filters with different porosities and a long service life. They are used to purify oil and liquid fuels and can withstand elevated temperatures.

In order to improve the microstructure uniformity of the alloy, sintering was performed in two steps. At the first step, the current density was increased with an increment of 56 A/cm^2^ up to *j* = 397–624 A/cm^2^ for 30 s. At the second step, sintering was conducted at *j* = 624 A/cm^2^ for 45 s, the other parameters remaining constant (*ω* = 2.6 kHz, *p* = 8,5488 kg/cm^2^). A homogeneous microstructure of the sintered alloy produced from a Cu-Ni powder mixture (50/50 wt %) with particles smaller than <40 µm is achieved in 45 s, while 4 h are needed to homogenize this alloy by annealing at a constant temperature of 1050 °C [5].

During sintering of Ni-Mo (80/20 wt %), Ni-based solid solutions form at lower energy input (*E* = 52.3 ×·10^5^ A^2^∙s/g∙cm), while at a higher energy input, complete dissolution of Mo in Ni takes place (*E* = 77.8 ×·10^5^ A^2^∙s/g∙cm) in 90–105 s [5].

#### 3.1.4. Nb-Based Systems Processed by Electric Current-Assisted Consolidation

Electric discharge sintering of Nb was studied to produce materials for metal-oxide-semiconductor capacitors [49]. The anodes were fabricated using two facilities VIU-20 and VIU-50 at 13–15 and 30–33 kV, respectively. During sintering, in order to reduce the axial shrinkage, the punches were fixed. As the applied pressure increases, the contact conditions between the punch and the powder are improved, which reduces erosion of the punch surface. Electric discharge sintering is efficient for making a porous structure of Nb without any binders retaining its purity [42]. Owing to the pinch-effect, the powder does not adhere to the surface of the punches [31]. This makes it possible to produce samples with open porosity and high specific surface area. Porous Nb samples sintered by electric discharge sintering have a higher porosity compared to conventionally sintered samples.

#### 3.1.5. Cu-Based Systems Processed by Electric Current-Assisted Consolidation

Copper has been widely used to fabricate plain bearings due to its high thermal conductivity and good running-in ability, however, its strength is not high. The introduction of silicon carbide, which is inexpensive and easily available, into a copper matrix results in a significant increase in strength and hardness of the material. However, the electrical conductivities of these phases differ dramatically. The way their combination will sinter under electric current is not obvious.

Mechanical properties of materials obtained by electric discharge sintering of 80%Cu-20%Sn powders with particles less than 50 µm in size under different sintering regimes were studied [50]. Sintering was performed at a constant pressure in two steps: at the first step, a current density of 580 A/cm^2^ was applied; at the second step, the current density was equal to 710 A/cm^2^. The best mechanical characteristics were achieved when the duration of the first stage was τ_1_ = 30 s. The highest density (96.6%–97.8%) and hardness of 89–93 HRB was observed in samples sintered at the second step for τ_2_ = 30, 25, 20 and 15 s. The highest bending strength (σ_b_ = 37.8 ×·10^7^ N/m^2^) was found in the sample sintered for τ_2_ = 15 s, while the samples sintered for τ_2_ = 20, 25 and 30 showed a lower bending strength of 32.2·× 10^7^–33.9·× 10^7^ N/m^2^. The highest impact strength (α_н_ = 9.5 kJ/m^2^) was observed in the sample sintered for τ_2_ = 25 s; the other samples showed a much lower impact strength.

Sintering of a bronze powder (9%Al-4%Fe-Cu) obtained by grinding of metallic chips was presented by Raichenko *et al.* [64]. The sintered samples were of ring shape. The hardness of the sintered materials was close to that of the cast materials (770 MPa), but the microhardness was lower. The relative density of the sintered samples was 98%, the impact strength was 35–46 kJ/m^2^ and the tensile strength was 340–380 МPа. The electrical resistivity of the sintered materials was in the range of 0.129–0.138 Ohm∙mm^2^/m and close to that of the cast material (0.123 Ohm∙mm^2^/m), which is an indication of efficient sintering of the powder particles. Low ductility of the sintered materials was due to the presence of Al and iron oxide phases.

#### 3.1.6. Ti-Based Systems Processed by Electric Current-Assisted Consolidation

Titanium powders are widely used to fabricate porous permeable materials. However, long-length porous permeable parts are rather difficult to produce by powder metallurgy [40].

Electric pulse sintering was employed to obtain long-length samples from titanium powders having coarse particles with sizes in the ranges of 0.2–0.16 and 0.315–0.4 mm. Titanium powder of VT-9 grade was used containing Ti 86.7%–90.4%, Al 5.8%–7.0%, Mo 2.8%–3.8%, Zr 0.8%–2.0%, Si 0.20%–0.35% [38]. Low-quality samples were obtained after a single cycle of electric pulse sintering; therefore, several cycles were necessary. It was found that the height of the powder column should be controlled to avoid melting of the material in the middle of the sintered sample due to high electric resistivity of the regions located far from the punches. The height of the sample to diameter ratio should be in the (7–27):1 range; if it is set to be higher, the material will unavoidable melt in the longitudinal direction [40]. This ratio has to be retained during several cycles of electric pulse sintering. When titanium powder of VT-9 grade produced by atomization of cast electrodes is sintered, the dendrite structure is preserved, in which the content of the b-phase is higher in larger dendrites than in smaller ones [34,36,43]. This indicates that diffusion processes do not have sufficient time to occur due to rapid heat conduction into the sample volume. In order to make a porous part, electric pulse sintering was followed by sintering in a vacuum furnace. The maximum strength of the materials sintered from titanium powder of VT-9 grade was achieved in the sample sintered at applied pressures ranging from 10 to 20 MPa [45]. When the pressure was higher than 10 MPa, a dramatic decrease in electric resistance of the sample was observed, which prevented its melting in certain zones. Electric pulse sintering of titanium of VT-0 grade with particle sizes 250 and 50–100 µm was also studied [22]. An electric current density applied was 120 kA/cm^2^. An input energy of 2.0 ×·10^3^ A^2^s/(g∙cm) was necessary to initiate sintering with a single pulse. At input energies of 4.0 ×·10^3^–4.5 ×·10^3^ A^2^s/(g∙cm), fibers 20–150 µm in diameter formed throughout the sample volume. For efficient sintering in electric pulse sintering, the thickness of the skin layer should be larger than the sample dimensions while the pulse duration should be shorter than the time required to heat the powder particles to prevent their complete melting. At a stepwise input of energy into the sample, the formation of electric arc and gas evolution can be prevented.

#### 3.1.7. Tungsten Pseudoalloy Powders Processed by Electric Current-Assisted Consolidation

Tungsten pseudoalloys W-Ni-Fe are promising structural materials of enhanced mechanical performance. They are used as protective materials against radiation and as materials of welding electrodes. Spark Plasma Sintered W-Ni-Fe alloys possess better mechanical properties compared to alloys conventionally produced by liquid-phase sintering [5]. Sintering of the 95% W-3.5% Ni-1.5% Fe alloy with a particle size of 4–5 µm was preceded by pressing using a hydrostatic pressure of 50 MPa. SPS was performed in vacuum in an SPS-625 (SYNTEX Inc, Kawasaki, Japan) at temperatures of 900–1300 °C and pressures of 50–70 MPa. The sample was heated by electric pulses of 3.3 ms, the heating rate was varied from 50 to 300 °C/min. The optimal sintering temperatures at a heating rate of 100 °C/min and 50 °C/min were determined to be 1100 °C and 1200 °C, respectively. The maximum density of the sample was 99.7%. The maximum strength of the sintered materials was 2250–2500 MPa reached at a heating rate of 100 °C/min and sintering temperatures of 925–950 °C, ductility of the material not exceeding 0.5%.

#### 3.1.8. Intermetallic Powders Processed by Electric Current-Assisted Consolidation

FeAl and Fe_3_Al intermetallics can withstand working temperatures of up to 680 °C. They are strong and light-weight materials of high erosion and corrosion stability caused by the presence of aluminum oxides. There materials are also wear-resistant and inexpensive. They are used in conveyer rollers of hot-rolled steel strips, as resistance-heating elements and in the automotive industry to substitute for corrosion-resistant steels in exhaust systems [95]. However, the application of these intermetallics is limited due to their low plasticity, which can be improved to some extent through alloying with Co.

Fe_3_Al was synthesized in spark plasma sintering from a mixture of Fe and Al [95]. Sintering was carried out using a SPS-511S apparatus (Sumitomo Coal Mining, Tokyo, Japan) at a pressure of 50 MPa and a holding time of 5 min. Heating up to 1000 °C was conducted in 12 min. The sintered sample consisted of Fe_3_Al (84.9%) and α-Fe (15.1%). The absence of Al shows that at the optimal ratio of Al and Fe, the volume fraction of the intermetallic can be increased up to 100%. Samples alloyed with Co were sintered under the same conditions [96]. After SPS in the materials containing Fe, Co and Al, several phases, such as FeAl, Fe_3_Al, FeAl_2_ and FeCo increase the microstrains in the material [21,96,98] while γ-Fe and Co are responsible for an increased plasticity. Sintering using an apparatus made by Thermal Technology LLC 10–3 (USA) [21,98] was conducted at the following parameters: the applied pressure was 60 MPa; the sintering temperature was 1200°C; the dwell time at the maximum temperature was 30 min; the residual pressure was 0.8 × 10^−2^–3 × 10^−2^ Hg. Sintering was performed in a graphite die. The properties of the sintered materials are presented in Table 2.

**Table 2:** Properties of the sintered materials.

Phase composition	Density, g/cm^3^	Hardness, HV	Reference
FeAl (85.72%), Fe_3_Al (2.13%), γ-Fe (2.22%), Co (9.93%)	6.46	740 ± 15	[21]
FeAl_2_(80.1%), Fe_3_Al (4.8%), Cr (15.1%)	6.67	510 ± 15	[98]
CoFe (40.1%), Fe_3_Al (59.9%)	7.5	464 ± 15	[96]

Considering the phase composition of the sintered materials, the formation of intermetallics was concluded to proceed by a solid state mechanism.

Sintering of FeAl can be performed in the high-frequency plasma of low pressure, a better sintering uniformity is then achieved and a new phase composition is obtained [123].

Sintering of Cu-8 wt % Al intermetallics received particular attention. Lattice parameters of a Cu-Al alloy produced by casting and by electric discharge sintering followed by a 40 s-dwell was compared [60]. The determined lattice parameters were very close indicating a high homogeneity of the sintered material.

Electric discharge sintering of Al-Cu mixtures was studied for three compositions [124]. The first two mixtures contained aluminum particles 40 µm in size; a coarser aluminum powder with 500 and 315 µm particles was used in the third mixture. The same copper powder was used to prepare all three mixtures, its size not exceeding 40 µm. Sintering was performed at a current density of 900 A/cm^2^, a current frequency of 2750 Hz and a final pressure of 137 MPa. The electrical resistance of the samples was measured and their porosity was thus calculated. Microhardness and impact strength were also measured (Table 3).

**Table 3:** Composition, sintering conditions and properties of the sintered materials.

Sample number	Composition	Al particle size µm	Initial pressure *P*_1_, MPa	Final pressure *P*_2_, MPa	Time τ, s	Porosity, %	Hardness HB, kgf/mm^2^	Electrical resistivity ρ∙10^8^, Ohm/m	Thermal conductivity α_н_, kJ/cm^2^
1	Al + 33% Cu	40	98	137	60	9.7	110.0	4.6	0.53
			98	137	95	2.7	156.0	5.1	0.94
2	Al + 5.7% Cu	40	98	137	33	15.8	43.0	3.7	0.69
			98	137	45	1.9	61.3	5.8	0.91
3	Al + 5.7% Cu	500 and 350	98	137	45	21.2	22.1	19.4	0.06
			39	137	35	3.4	26.2	4.2	0.65
			39–16	137	25	7.8	27.2	6.9	8.9

XRD phase analysis of the sample sintered for 60 s showed that the sample contained Al and Cu as major phases. The CuAl_2_ was present as a minor phase and only very small amounts of Cu_3_Al_4_ were detected. In the sample of the same initial composition sintered for 95 s, the Cu_3_Al_4_ was not found. The microstructure of this alloy was typical of eutectic alloys with the inclusions of CuAl_2_ responsible for high microhardness. In the composition denoted (2) sintered for 33 s, the Al, Cu and Cu_3_Al_4_ phases were found. In the same alloy sintered for 45 s, Cu was fully dissolved in Al. The CuAl_2_ was present as thin layers located along the grain boundaries. When composition (3) was sintered at a pressure of 16 MPa, the material with the highest content of CuAl_2_ was obtained.

The accelerated formation of the alloy during the electric discharge sintering is explained by the destruction of A1_2_O_3_ films under the microflows of plasma as well as by circulation of liquid and gas phases at the inter-particle contacts.

When metallic powders are sintered by the methods described above, the sintering parameters should be carefully chosen, namely, current, voltage, pressure, sintering time or the number and duration of the pulses. Along with the sintering parameters, heat dissipation into the punches should be taken into account as well as adherence of the powders to the punch surfaces, and non-uniform heating of the die and punches. Flows induced by electric current in a molten metal significantly influence the microstructure of an alloy. The composition and particle sizes of the powders should be selected depending on the possible application of the sintered material.

### 3.2. Ceramic Materials Processed by Electric Current-Assisted Consolidation

Consolidation of ceramic materials by electromagnetic field-assisted methods has been widely studied in the USSR and post-soviet countries.

Processing of ceramic materials is challenging due to high-temperature processes involved. Studies of sintering of ceramics have the potential of finding the optimal processing conditions to obtain ceramic materials with desired properties. This part of the review deals with the major issues of sintering of ceramic materials by means of electric current, which creates conditions of fast heating and sintering accompanied by limited grain growth. The optimal sintering conditions for consolidation of zirconia and alumina ceramics are presented. Particular attention is directed to titanium nitride ceramics as well as composite ceramics. Sintering of metal-ceramic mixtures is also discussed.

#### 3.2.1. Processing of ZrO_2_-Ceramics

Sintering of zirconia ceramics is an interesting case, as this ceramic material has a high electrical conductivity dependent on the temperature. In order to control the sintered microstructure and properties of the sintered material, the parameters of the initial powders need to be properly selected [107].

**Figure 29:**
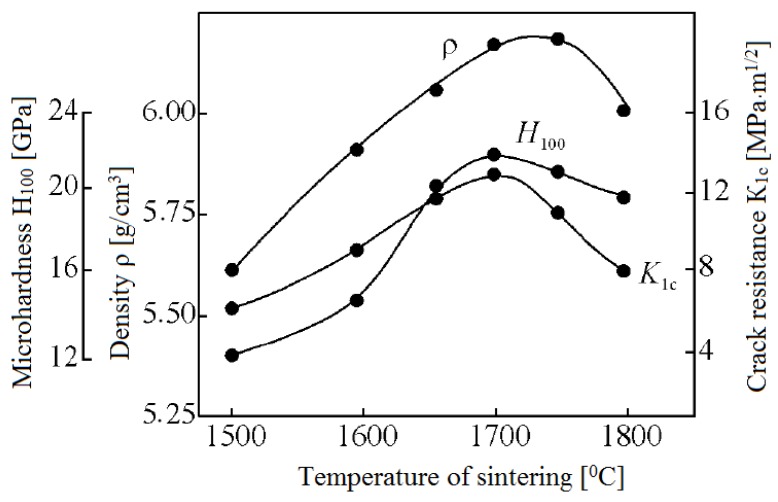
Microhardness Н_100_, density ρ and crack resistance К_1с_ of zirconia ceramics sintered in the ammonia plasma of a glow discharge.

Scientists of Tomsk State Polytechnic University studied sintering of zirconia and alumina. Slosman and Matrenin [25] sintered zirconia stabilized by yttria (3%) in the ammonia plasma of a glow discharge. The samples were held for 0.25–2 h at 1500–1800 °C. The properties of the sample sintered for 2 h are shown in Figure 29, which demonstrates that the maximum density, crack resistance and microhardness are achieved in the sample sintered at approximately 1700 °C.

A study was also carried out, in which sintering in a vacuum furnace at a pressure of 0.1 Pa and in air plasma conducted at the same temperatures and for the same durations were compared. A noticeable reduction in porosity was observed after sintering in the ammonia plasma of a glow discharge; the microhardness and crack resistance was higher than that exhibited by the samples sintered in vacuum or in air plasma.

Electric current-assisted consolidation of ZrO_2_-3%Y_2_O_3_ was also studied by researchers of Kharkov Institute of Physics and Technology [125].

**Figure 30:**
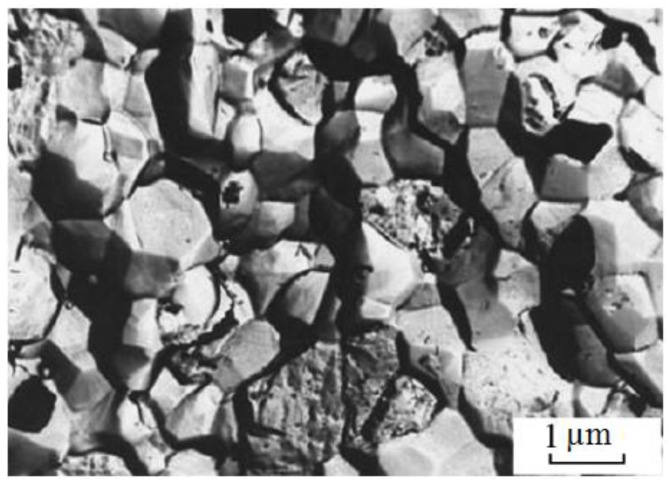
Microstructure of the ZrO_2_-3% Y_2_O_3_ sample sintered at 1450 °C and 40 MPa.

The samples to be sintered were shaped by one-sided uniaxial pressing at a pressure of 130 MPa. The pressed samples were sintered at 1450 °C and 40 MPa for 15, 30 and 45 min. The optimal conditions for densification were reached for the samples sintered for 30 min, the material density reaching 6.00–6.07 g/cm^3^ (the density of the pressed samples was in the range of 2.5–2.6 g/cm^3^). Figure 30 shows that the microstructure of the sintered material is quite uniform and fine-grained.

In 2010, Matrenin and co-workers from Tomsk State Polytechnic University and Tyumen State Oil and Gas University published another article on sintering of ZrO_2_-based ceramics [126]. In that work, sintering of the ZrO_2_-based ceramics modified by Al nanopowders was studied.

The initial powders had a spherical morphology, the particle diameters ranging from 0.1 to 1 μm. The powder particles consisted of crystallites 20–50 nm in size; the particle size of Al nanopowders was 50–500 nm.

Prior to sintering, the powders were pressed at a pressure of 400 MPa and then ground in a planetary mill, an operation required to increase densification of the material during sintering. Consolidation of the powders was carried out by sintering in the plasma of a glow discharge, in air and in a high-temperature resistance furnace.

The sample, to which Al nanopowders were added (5%), retained its shape after sintering in air, but deep cracks formed. As the content of Al increased up to 10%–20%, the density of the sintered material increased, its shape was well retained and no cracks were observed. However, all samples were rather porous, the volume of pores being 25% and larger. The samples were heated up to 1600 °C for 6 h, kept at this temperature for 1 h and then cooled down to room temperature during 8 h.

The same samples were sintered in the ammonia plasma of a glow discharge at 1600–1650 °C. The sample containing 20% of the Al nanopowder showed a layered structure consisting of three layers of different microhardness and porosity. The authors suggest that this is related to a high temperature gradient from the surface of the sample into its volume. A conclusion was made that during sintering of zirconia containing Al nanopowder additives in the ammonia plasma, a reaction of self-propagating high-temperature synthesis of zirconium nitride is activated.

Gevorkyan *et al.* [127,128] investigated electroconsolidation of ZrO_2_ stabilized by Y_2_O_3_ (3 wt %). The applied pressure was 40–45 MPa. The particle size of the initial powder was 20–30 nm. The best microstructure characteristics—density, uniformity, grain size—were obtained after sintering at 1050 °C. The grain size in this material was 250–300 nm.

#### 3.2.2. Processing of Al_2_O_3_-Ceramics

**Figure 31:**
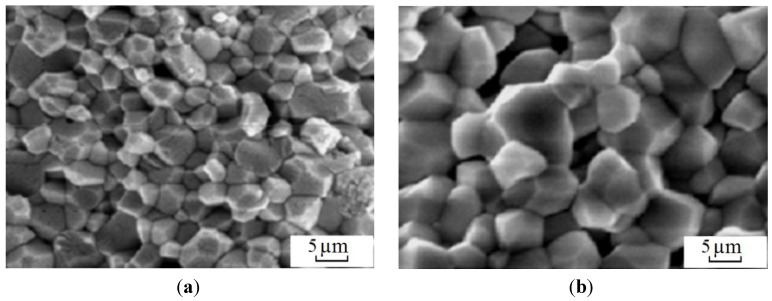
Microstructure of the sintered Al_2_O_3_: (**a**) at 1100 °C; and (**b**) at 1400 °C.

Gutsalenko and Gevorkuyan [106] studied electric current-assisted sintering of alumina Al_2_O_3_. They conducted hot-pressing with direct heating by electric current. The grain size of the initial alumina nanopowder was 60–80 nm. The samples were held at 1400 °C for 2 min; different heating rates were used. It was found that independently of the heating rate, the grain size of the sintered material lies in the submicron range and does not significantly change. Under the lowest heating rate used (50 °C/min) the grains grew to a size of 6–9 μm (Figure 31). The density of the sample sintered at 1400 °C was 95%.

**Figure 32:**
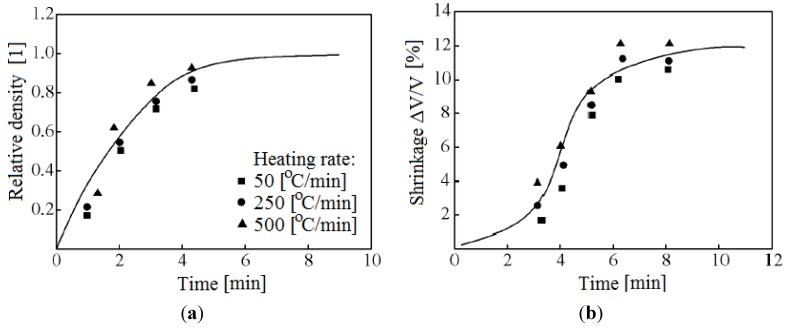
(**a**) The density and (**b**) shrinkage of Al_2_O_3_ *vs.* sintering time at different heating rates. The pressure was gradually increased from 10 MPa at 400 °C to 45 MPa at 1400 °C.

The densification rate of the samples was shown to be largely independent of the heating rate (Figure 32).

Activated sintering of alumina containing additions of Al nanopowders and Al_2_O_3_ was also considered [126]. The particle size of the initial powder was 0.1–1 μm while the crystallite size was 20–50 nm. The particle size of the Al nanopowder was 50–500 nm. After mixing for 1 h, the powders were pressed using a steel die. Sintering was conducted in a glow discharge. For the transformation of γ-Al_2_O_3_ into α-Al_2_O_3_ to occur, annealing of a coarse Al_2_O_3_ powder in air was carried out.

**Figure 33:**
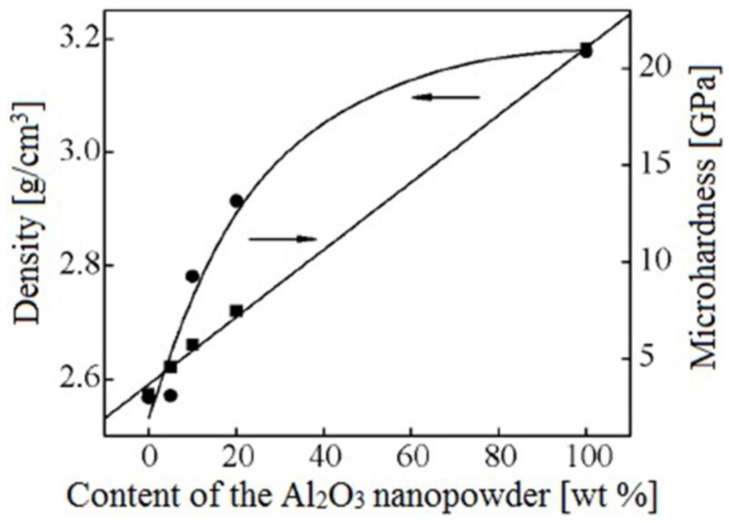
The dependence of density and microhardness of the corundum ceramics sintered from a coarse α-Al_2_O_3_ on the content of the Al_2_O_3_ nanopowder in the mixture.

The variation of density and microhardness of the corundum ceramics sintered from a coarse α-Al_2_O_3_ with the content of the Al_2_O_3_ nanopowder in the mixture is shown in Figure 33. As can be seen from the plots, the density and microhardness of the corundum ceramics increase with increasing Al nanopowder content, the density increasing linearly.

At the same time, the authors point out that the microhardness measurements may have not been totally correct. This is related to the cases when the indentations were made in the vicinity of pores, the number and size of which decreased with increasing Al_2_O_3_ nanopowder content.

**Figure 34:**
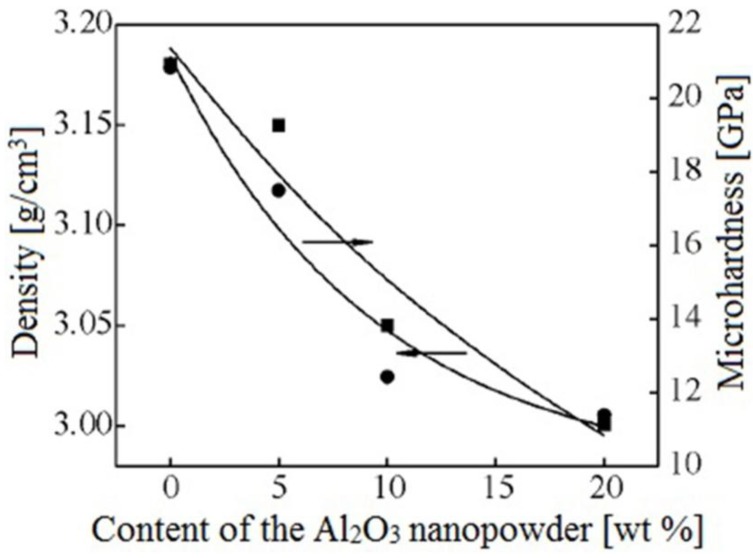
The dependence of the density and microhardness of the samples sintered from Al_2_O_3_ nanopowders on the content of the Al nanopowder.

Figure 34 shows the dependence of the density and microhardness of the samples sintered from the Al_2_O_3_ nanopowders on the content of the Al nanopowder introduced into the mixture.

A decrease in microhardness and density with increasing content of the Al nanopowder is due to oxidation of Al leading to the formation of pores in the sintered material.

**Figure 35:**
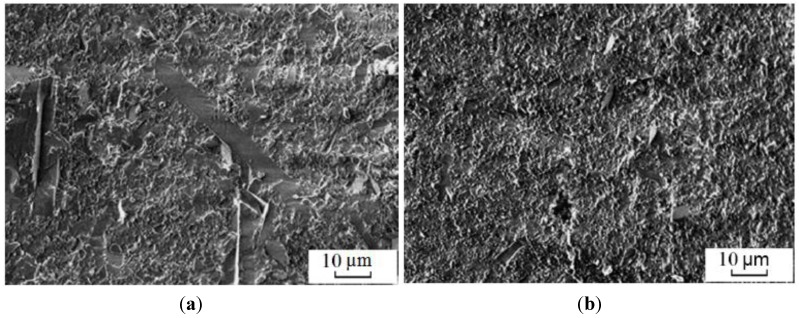
Surface of the alumina-based sintered compacts: (**a**) 1400 °C, 15 min; and (**b**) 1350 °C, 5 min.

Petrova *et al.* [99] sintered nanostructrured alumina by SPS at a pressure of 60 MPa and at different temperatures. The samples were of cylindrical shape with a height of 2 ± 0.2 mm and a diameter of 19.8 ± 0.2 mm. The authors show that the surface morphology of the sintered compacts depends on the sintering conditions (Figure 35).

As the sintering temperature increases up to 1400 °C, the microhardness of the sintered material significantly increases relative to that sintered at 1200 °C (Table 4). The density values also reach maximum at 1400 °C. As the temperature further increases, the density of the material remains practically unchanged while its microhardness decreases. The optimal parameters of the SPS-process for alumina have been found: *T* = 1350 °C, holding time *t* = 5 min, pressure *P* = 60 MPa.

**Table 4:** Microhardness and density of the SPS-ed alumina.

No.	SPS conditions		Mechanical properties	
	Temperature, °C	Time, min	Microhardness HV	Density, g/cm^3^
1	1200	10	180	2.40
2	1300	10	930	3.60
3	1400	15	2035	3.92
4	1500	15	1669	3.91
5	1350	5	2046	3.84

#### 3.2.3. Processing of TiN-Based Ceramics

**Figure 36:**
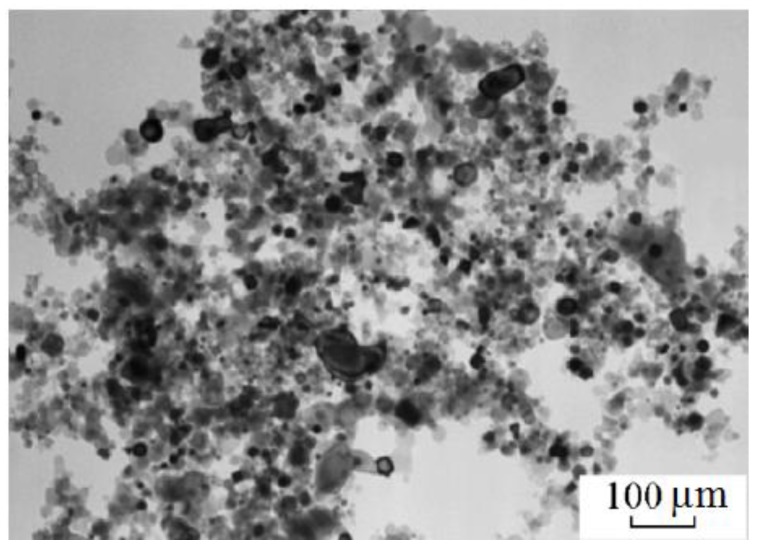
TEM image of the starting TiN powder.

**Figure 37:**
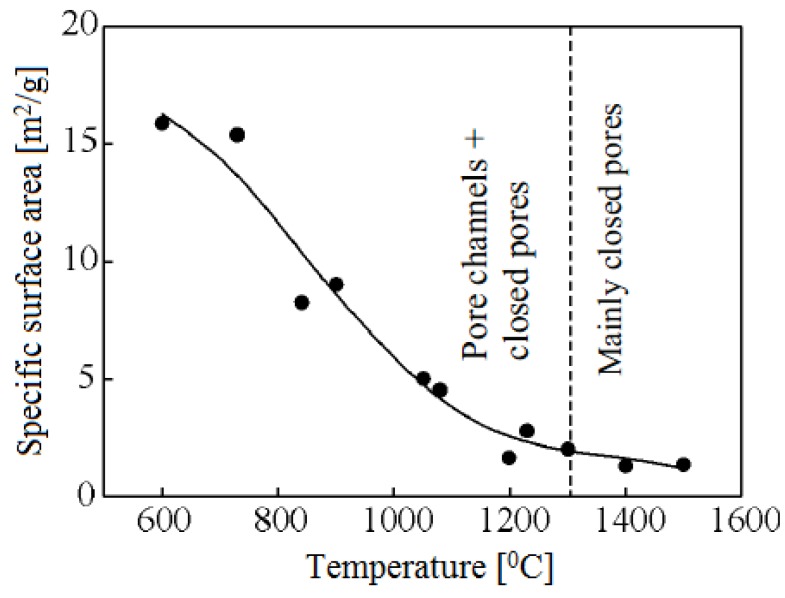
Specific surface area of sintered polycrystalline TiN as a function of sintering temperature.

Spark plasma sintering of nanocrystalline titanium nitride was studied by the scientists of Frantsevich Institute for Problems of Materials Science, National Academy of Sciences of Ukraine [92]. The size of the powder was 12–25 nm (Figure 36). Sintering was carried out at a constant heating rate in a temperature interval of 600–1500 °C at a pressure of 80 MPa. The shrinkage of the sample started at 800 °C while in the interval between 800 and 1200 °C, a significant reduction of the specific surface area was observed (Figure 37).

**Figure 38:**
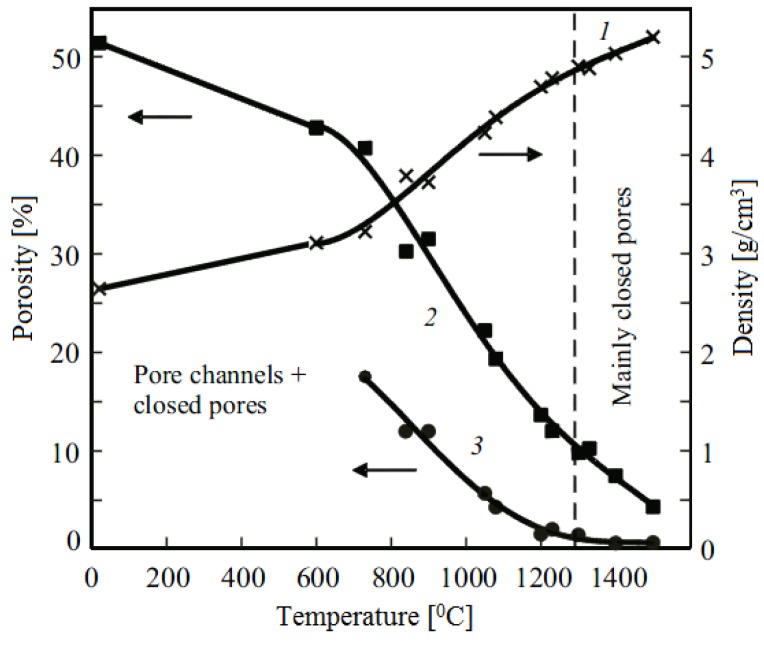
Density (**1**) and porosity (**2** and **3**) of TiN sintered at different temperatures: 2—total porosity and 3—pores to 300 nm.

Sintering resulted in an increase of density from 2.64 g/cm^3^ before sintering to 5.2 g/cm^3^ in the compact sintered at 1500 °C (Figure 38).

Substantial shrinkage was observed as the temperature increased up to 1200–1250 °C. Upon an increase in temperature above 1300 °C, reduction of the pore size was more difficult and was accompanied by grain growth and a dramatically decreased densification rate.

Electron microscopy has shown that after sintering at 1200 °C, the sample contains crystallites about 100 nm in size; as the sintering temperature increases up to 1300 °C, the grains acquire a clear faceted morphology and grow to a size of 1 µm. Polished samples sintered at 700–800 °C reveal agglomerates of spherical particles 1–5 μm in size.

**Figure 39:**
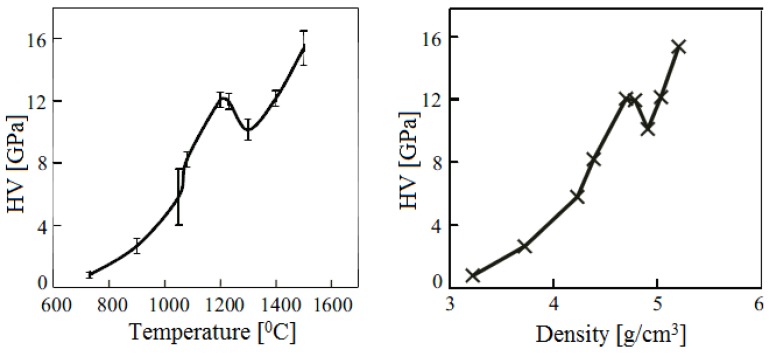
Vickers microhardness of sintered TiN samples.

A noticeable drop in the microhardness is observed in the sample sintered at 1300 °C, while the microhardness of samples sintered at higher temperatures is higher (Figure 39). The observed effect is explained by the simultaneous tenso-effect and a change in the chemical composition.

Based on these results, the temperature range of 1200–1300 °C was selected as optimal to sinter TiN while avoiding significant grain growth and achieving maximum densification and good mechanical properties.

#### 3.2.4. Processing of Ceramic Composites

**Figure 40:**
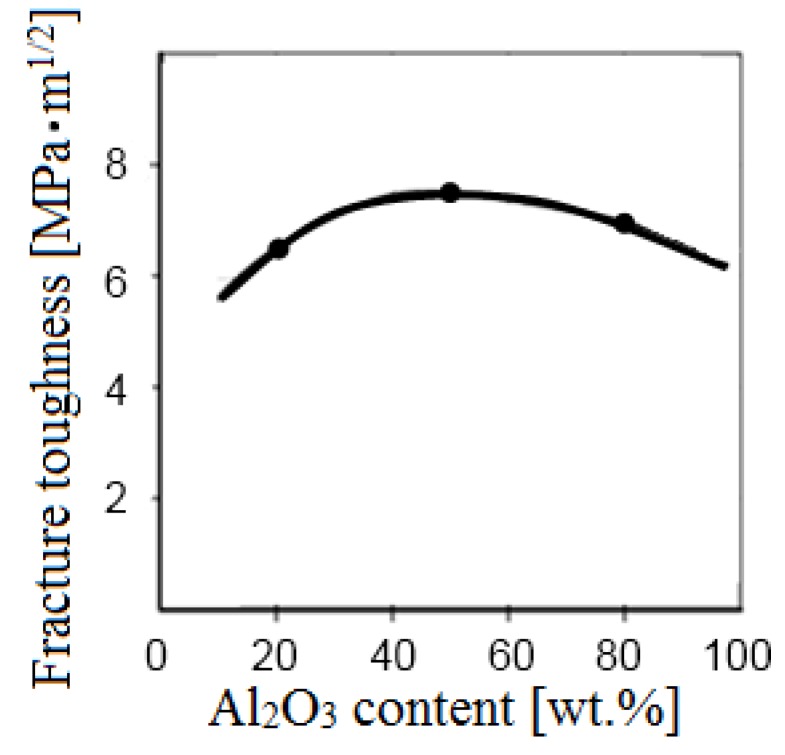
The fracture toughness of the sintered Al_2_O_3_-WC materials *vs.* the Al_2_O_3_ content (wt %).

**Figure 41:**
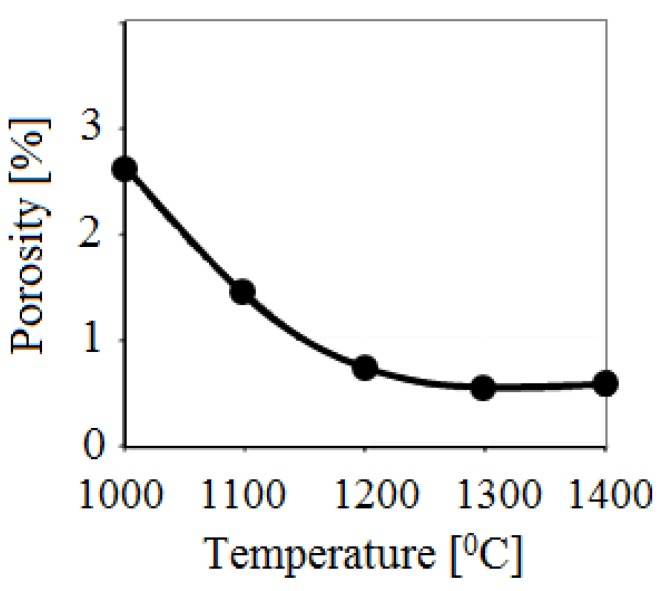
The dependence of the porosity of the Al_2_O_3_-WC sintered materials (50/50 wt %) on the sintering temperature.

Gevorkyan and Gutsalenko studied the possibilities of improving the properties of the Al_2_O_3_-WC ceramic composites as promising tool materials [110]. The initial powders were nanopowders of Al_2_O_3_ with a grain size of 60–80 nm and nanopowders of WC with a grain size of 40–70 nm. The powders were mixed in a ratio of 50/50 wt %. Sintering was performed by hot-pressing, during which electric current passed through the graphite dies at an applied pressure of 45 MPa. The samples were heated up to 1600 °C at a rate of 150–200 °C/min. The samples showed fracture toughness in the range of 8–12 MPa m^1/2^ and hardness НRA of 91–93. As can be seen from Figure 40 and Figure 41, the Al_2_O_3_-WC composite (50/50 wt %) sintered at 1150 °C shows the highest fracture toughness and very low porosity (1%).

**Figure 42:**
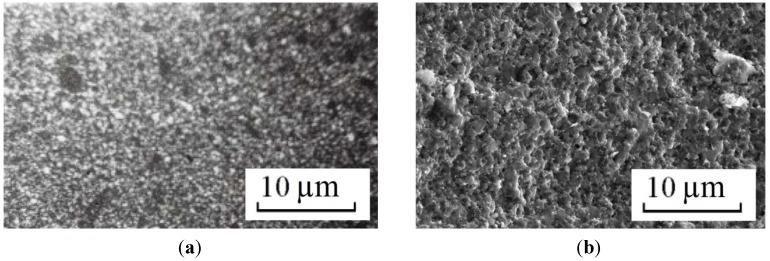
Microstructure (**a**) and fracture surface (**b**) of the sintered Al_2_O_3_-WC composite (50/50 wt %).

After sintering at 1500 °C, the density of the Al_2_O_3_-WC sintered materials (50/50 wt %) reaches 100%. Figure 42 shows the microstructure and fracture surface of the sintered composite [24].

The obtained results are compared with the properties of a well-known ceramic material—VOK-71 (71% Al_2_O_3_ + 20% TiC + 9% ZrO_2_)—which has a density of 4.2–4.27 g/cm^3^. The density of the obtained ceramics is higher (5.96 g/cm^3^) making it a more promising material for cutting plates compared to those made of Al_2_O_3_-based materials containing additions of TiC [24].

Dyatlova of Virial, Saint-Petersburg studied the formation of Al_2_O_3_-ZrO_2_ ceramics during sintering. The 58.6% Al_2_O_3_-38.3% ZrO_2_-3.1% Y_2_O_3_ (wt) composition was selected [129]. The as-synthesized composite powder consisted of agglomerates 150 nm in size composed of smaller particles 10–20 nm in size. Sintering was carried out by three methods: conventional pressure-less sintering, hot-pressing and spark plasma sintering.

In conventional sintering, the sample was held for 2 h at a temperature of 1550 °C. The optimal conditions of hot-pressing and SPS were experimentally found; the optimal temperature during hot-pressing was found to be 1550 °C while that for the SPS was 1350 °C. In both cases, the heating rate was 200 °C/min and a pressure of 35 MPa was applied.

**Figure 43:**
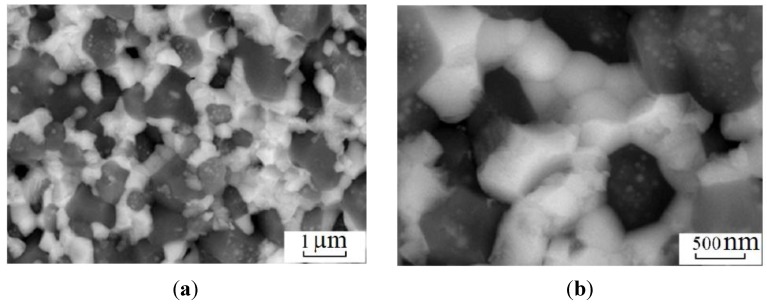
Microstructure of the sintered Al_2_O_3_-ZrO_2_ consolidated by hot-pressing at *T* = 1500 °C: (**a**) 14000×; (**b**) 33000×.

In the hot-pressed sample, the grain size of zirconia was 0.4–0.5 µm while that of alumina was 0.8–0.9 µm (Figure 43).

In the SPS-ed composite, the grain size of ZrO_2_ was 0.4–1.0 µm while the grain size of Al_2_O_3_ was 0.7–2.0 µm. After the SPS, the microstructure of the Al_2_O_3_-ZrO_2_ ceramics did not change significantly; however, during hot-pressing the microstructure of the ceramic composite markedly degraded.

Mechanical properties of the compacts sintered by hot-pressing are superior to those of the conventionally sintered compacts (Table 5).

**Table 5:** Comparison of the mechanical properties of Al_2_O_3_-ZrO_2_ ceramics sintered by different methods.

Sintering mode	*T*, °C	σ_b_, MPa	HV, GPa	*K*_1C_, MPa·m^½^
Conventional sintering	1550	730	13	7
Hot-pressing	1500	1050	17	8
SPS	1350	1020	17	8

Annenkov and Ivashutenko of Tomsk State Polytechnic University studied the preparation of nanocrystalline oxide ceramics by high-energy density technologies [130]. The advantages of several methods were discussed including those of magnetic-pulse compaction based on the studies conducted in Tomsk State Polytechnic University, Institute of Electrophysics, Ural Branch of the Russian Academy of Sciences and Siberian Chemical Plant.

Romanova *et al.* [23] of Kazan State Technical University synthesized ceramic materials from the mixtures of MgO, Al_2_O_3_ and SiO_2_ oxides taken in the ratio 2:2:5. Sample 1 contained silicon dioxide as a fine powder of high purity; sample 2 contained nanosized silicon dioxide synthesized by a chemical method.

**Figure 44:**
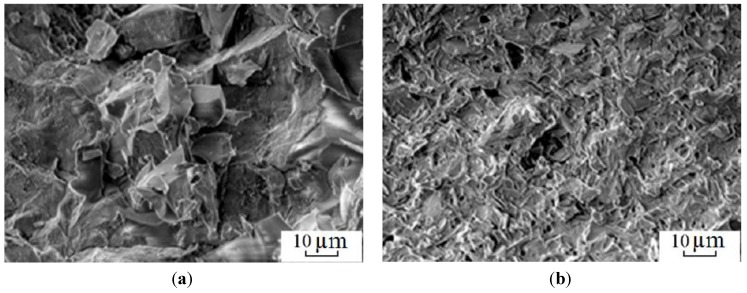
Fracture surface of the samples sintered from the MgO-Al_2_O_3_-SiO_2_ mixtures: sample 1 (**a**) and sample 2 (**b**).

The mixtures were pressed at a pressure of 60 MPa. Then, sintering was conducted in vacuum for 30 min at 1200 °C. Figure 44 shows fracture surfaces of the sintered materials. The microstructure of sample 2 is rather uniform, the crystallite size ranging from 1 to 5 µm (Figure 44 b). The fracture surface of sample 1 shows large layered fragments 10–30 µm in size, a glass phase and quartz crystallites of 5–10 µm (Figure 44 a).

In 2010, scientists from Dagestan State University and Dagestan State Technical University investigated consolidation of SiC-AlN ceramics by electric pulse sintering [55,56].

The initial powders were SiC and AlN synthesized by a plasmochemical route. An average particle size was 0.4 µm, about 1.25% of the total weight of the powder was composed of particles about 2 µm in size. Sintering resulted in the formation of dense SiC-AlN ceramics.

Sintering of ceramic materials containing titanium diboride was the subject of research in Frantsevich Institute for Problems of Materials Science, National Academy of Sciences of Ukraine. This ceramic material is attractive in view of its thermochemical and mechanical properties. However, high melting temperature of these ceramics makes it very challenging to sinter the material and not induce grain growth as a prolonged exposure to high temperatures is required.

In 2007, reaction sintering of TiN/TiB_2_ composites was studied by researchers of Frantsevich Institute for Problems of Materials Science, National Academy of Sciences of Ukraine [60]. Table 6 shows the density and mechanical properties of the TiN/TiB_2_ composites sintered at 1500 °C from powder mixtures synthesized by different techniques. The starting TiH_2_ has a cubic lattice (CaF_2_ structural type) and TiH*_x_* tetragonal.

**Table 6:** Properties of sintered TiN/TiB_2_ composites.

Initial powder	X-ray phase analysis	Relative density γ, %	Microhardness HV, GPa	Fracture toughness *K*_1c_, MPa·m^½^
TiH*_x_* + BN	TiN + TiB_2_	96.50	23.38	4.71
TiH_2_ + BN	TiN + TiB_2_ + BN(VW)	36.33	25.45	3.27
Ti + BN	TiN + TiB_2_ + BN(VW)	90.83	21.36	3.02

Samples sintered from powders containing metallic titanium are rather non-uniform and show a broad grain size distribution. The material consists of coarse grains 80–100 µm in size, which are composed of subgrains of 1–4 µm. Samples sintered from titanium hydrides are of uniform microstructure and contain equiaxed grains with an average size of 1 µm.

**Figure 45:**
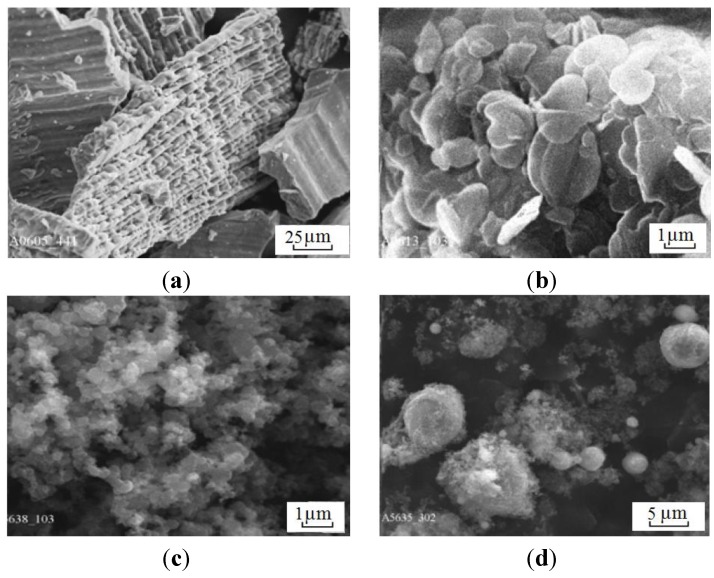
Morphologies of the initial powders: (**a**) TiH_2_, (**b**) BN, (**c**) B and (**d**) TiN.

Petukhov [62] continued investigations in this area. It was found that it is possible to synthesize the TiN-TiB_2_ composites by electric discharge sintering without any additives. The density of the composites varied from 64% to 99%. Morphologies of the initial powders are shown in Figure 45.

The powder mixtures contained large agglomerates of TiH_2_ and layers of BN. The size of the agglomerates varied from 1 to 10–20 µm. Sintering was performed using two methods: by electric discharge sintering using ERAN-2/1 facility for 180 s at a pressure of 60–80 MPa and at a heating rate of 28–42 °C/s and by SPS (Sumitomo, Tokyo, Japan) at a pressure of 40 MPa, at a heating rate of 2–4 °C/s for 480–670 s.

Materials containing 36–60 wt % TiB_2_ sintered using ERAN-2/1 facility show good mechanical behavior: hardness of 19.7–25.4 GPa (load 100 g); fracture toughness of 5.4–5.8 MPa·m^1/2^ (load 20 kg). During sintering in ERAN-2/1 (Frantsevich Institute for Problems of Materials Science, Kiev, Ukraine), thanks to a higher heating rate, BN is decomposed by just evolved hydrogen and the TiN/TiB_2_ composite starts forming. When the content of TiB_2_ increases up to 80%, intense heating takes place leading to the damage of the graphite die.

**Figure 46:**
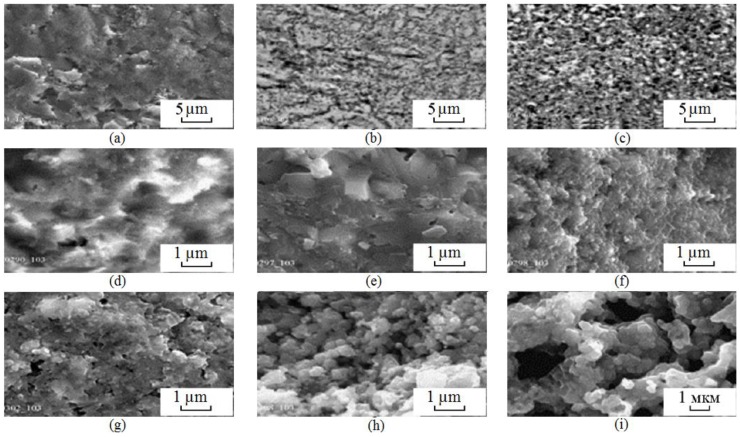
Microstructures of the sintered TiN/TiB_2_. Electric discharge sintering: (**a**) 20% TiB_2_; (**b**) 36% TiB_2_; (**c**) 60% TiB_2_. SPS: (**d**) 20% TiB_2_, *Ts* = 1400 °C; (**e**) 20% TiB_2_, *Ts* = 1500 °C; (**f**) 36% TiB_2_, *Ts* = 1400 °C; (**g**) 36% TiB_2_; *Ts* = 500 °C; (**h**) 60% TiB_2_, *Ts* = 1400 °C; (**i**) 80% TiB_2_, and *Ts* = 1400 °C.

Figure 46 shows that samples, sintered by electric discharge sintering, exhibit a fine-grained and uniform microstructure with an average grain size of 500–700 nm. Samples sintered by SPS at a lower heating rate have grains of about 1 µm and a rather non-uniform microstructure. In addition, those samples show some porosity (Figure 46, d–i). With increasing sintering temperature, the density of the SPS-ed materials increase, but other properties do not improve. At the same time, as the content of TiB_2_ increases, the porosity and non-uniformity of the microstructure of the sintered material increases.

**Figure 47:**
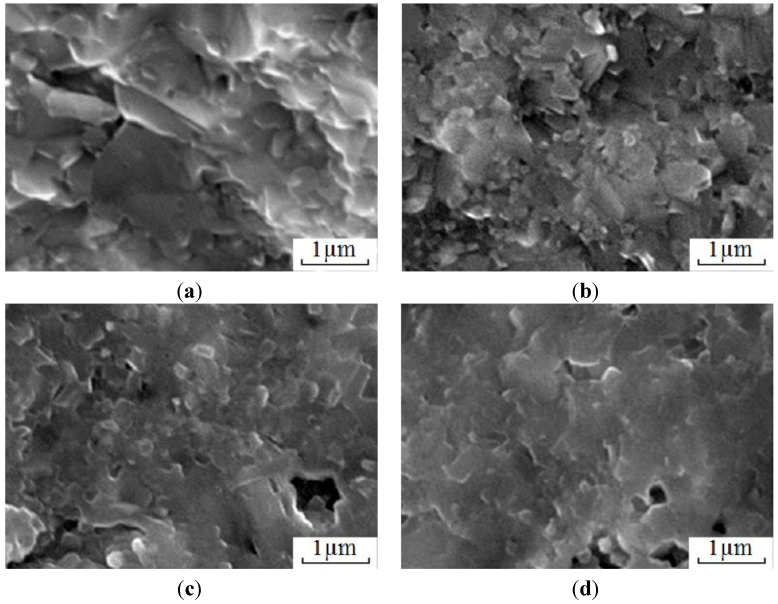
Microstructures of the sintered composites: (**a**) 20% TiB_2_ + 80% TiN; (**b**) 36% TiB_2_ + 64% TiN; (**c**) 60TiB_2_ + 40% TiN; and (**d**) 80% TiB_2_ + 20% TiN.

Another study of Frantsevich Institute for Problems of Materials Science, National Academy of Sciences of Ukraine was also devoted to the SPS of the TiN-TiB_2_ composites [113]. Initial powders used for the synthesis were TiH_2_, BN, B and TiN. Sintering was carried out for 3 min at a temperature of 1600 °C. The total cycle duration was 12–17 min with a heating rate of 112–300 °C/min. Four compositions were sintered: 20% TiB_2_ + 80% TiN; 36% TiB_2_ + 64% TiN; 60% TiB_2_ + 40% TiN; 80% TiB_2_ + 20% TiN (wt). The microstructures of the sintered materials are presented in Figure 47.

In the 20 wt % TiB_2_ + 80% TiN composite, the average grain size was 1–3 µm while in the 36% TiB_2_ + 64% TiN composite it was 200–300 nm. In the 60%TiB_2_ + 40% TiN and 80% TiB_2_ + 20% TiN compositions, the grain size was 300–600 nm.

The sample of the highest density is the 36% TiB_2_ + 64% TiN composite obtained at a heating rate of 225 °C/min (Table 7).

**Table 7:** Density and mechanical properties of the sintered TiB_2_-TiN composites.

Composition, wt %	Relative density, %	Hardness HV, GPa	
		Load, *P* = 0.1 kgf	Load, *P* = 2 kgf
20TiB_2_-80TiN	96.7	19.5(±0.58)	18.0 (±0.58)
36TiB_2_-64TiN	97.7	21.0 (±0.98)	20.0 (±0.59)
60TiB_2_-40TiN	95.3	22.0 (±1.78)	19.5 (±0.64)
80TiB_2_-20TiN	94.2	16.5 (±3.15)	15.9 (±0.66)

Scientists of Ioffe Physical Technical Institute, Russian Academy of Sciences in collaboration with researchers from Max Planck Institute for Chemical Physics of Solids, Germany were the first to show that high-strength refractory coatings can be deposited on Al_2_O_3_ by SPS [131].

**Figure 48:**
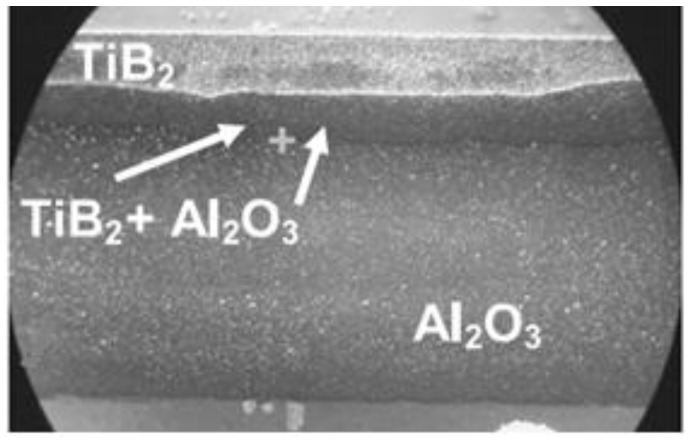
General view of layers obtained as a result of SPS of Al_2_O_3_ and TiB_2_.

In order to coat alumina with titanium diboride, the initial components TiB_2_ and Al_2_O_3_ were mixed in the ratio of 1:1. Coatings of boron suboxide were obtained from a mixture of B and B_2_O_3_. The samples of the first series were sintered in a graphite die at 1500 °C for 30 min. In order to minimize cracking upon cooling, the sample was slowly cooled down to 600 °C over 2 h. A layered structure formed after sintering consisting of a TiB_2_ layer and a composite Al_2_O_3_ + TiB_2_ layer on the Al_2_O_3_ substrate (Figure 48). The microhardness of the layers was 43 GPa and 27–36 GPa, for the TiB_2_ layer and the composite layer, respectively. The microhardness of the substrate was measured to be 25 GPa.

The samples of the second series were heated up to 1360 °C and held for 10 min. Cooling was conducted in the manner described above. In the samples of the second series, a layered structure also formed, however, the coating B_6_O detached from the substrate. An explanation for this effect suggested by the authors was a large difference in the microhardness of the coating layer and that of the substrate (65 and 25 GPa) and associated brittleness.

In Frantsevich Institute for Problems of Materials Science, National Academy of Sciences of Ukraine, electric discharge sintering was used to produce layered refractory materials TiN-AlN and B_4_C-TiB_2_ [87]. Initial TiN, AlN, B_4_C and TiB_2_ powders were mixed to produce the following compositions: TiN-20 wt % AlN, TiN-35 wt % AlN and B_4_C-70 wt % TiB_2_. The particles size of the initial powders observed by means of scanning and transmission electron microscopy was 30 nm for TiN, 50 nm for AlN, 5 μm for B_4_C and 3 μm for TiB_2_.

Electric discharge sintering was carried out at a pressure of 80 MPa, a direct current of 1.1 kA and an alternating current of 0.3 kA. By changing the current, the sintering temperatures were varied between 1650 and 1700 °C for the TiN-AlN composites; the B_4_C-TiB_2_ composites were sintered at 1500 °C. The total process time was 180–240 s.

In the TiN-AlN composite, the grain size of the TiN phase was 500–1000 nm and that of the AlN phase was 200–300 nm. The densities of the samples containing 20 and 35 wt % AlN were 94.7% and 95.9%, respectively.

The relative density of the B_4_C-TiB_2_ composite was 98%, the grain size of the B_4_C phase was 7–10 μm while that of TiB_2_ was smaller than 5 μm.

**Figure 49:**
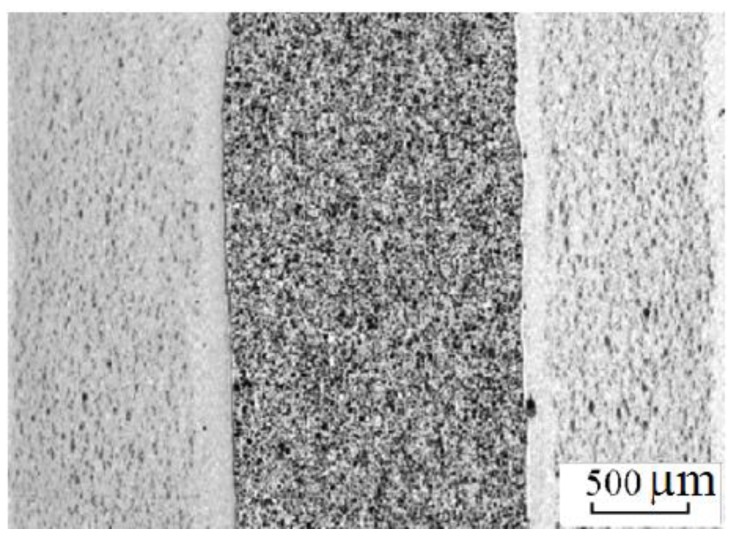
Microstructure of the layered TiN-20AlN/B_4_C-TiB_2_/TiN-35AlN composite.

**Figure 50:**
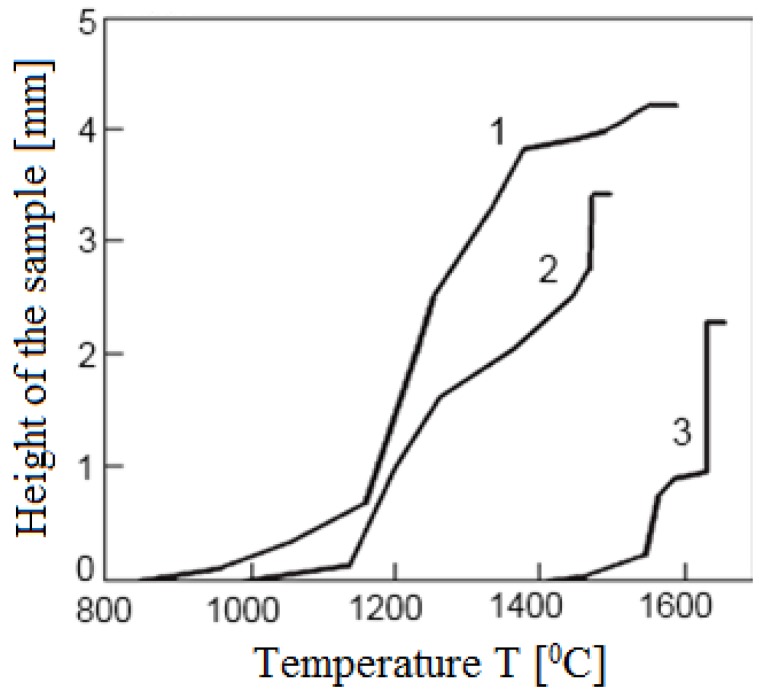
Variation of the sample height with the temperature during electric discharge sintering: 1—multi-layered composite; 2—TiN-20AlN; and 3—B_4_C-TiB_2_.

**Figure 51:**
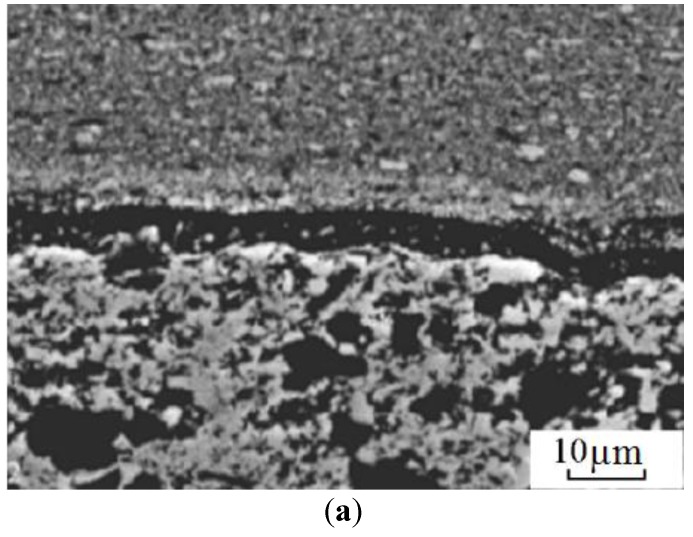

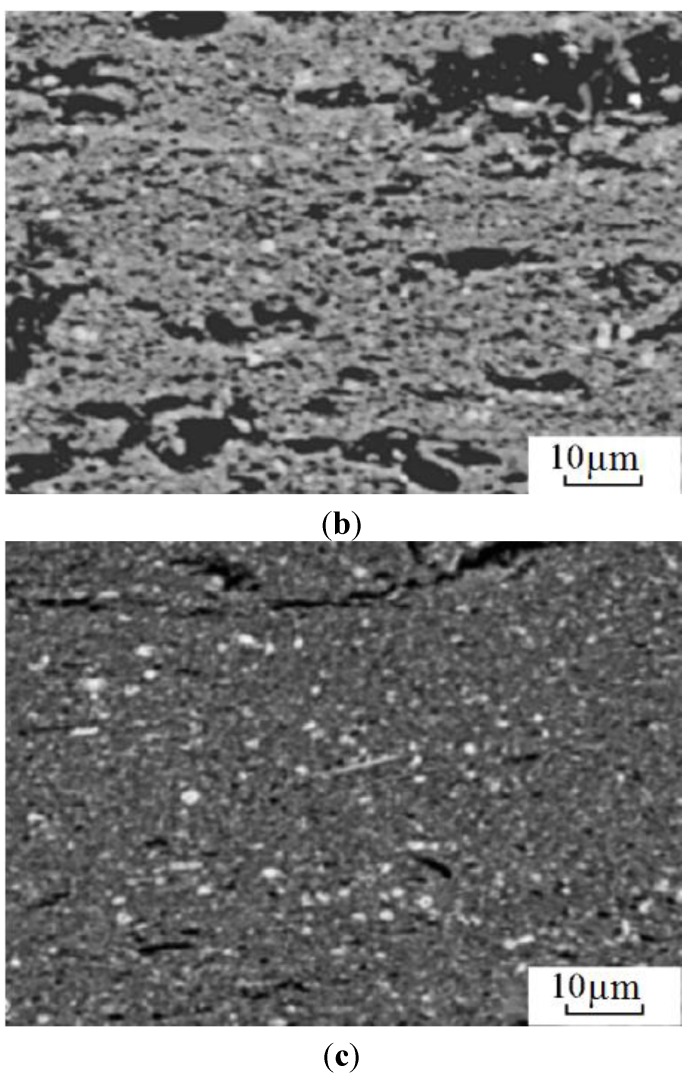
Microstructure of the TiN-35AlN layer in a 3-layer structure: (**a**) near the boundary with the B_4_C-TiB_2_ layer; (**b**) in the center of the layer; and (**c**) near the surface of the sample.

Sintering of two layered structures was performed, one of them was TiN-20AlN/B_4_C-TiB_2_ and the other was TiN-20AlN/B_4_C-TiB_2_/TiN-35AlN (Figure 49). Densification of the layered material begins earlier and proceeds faster than in materials of the layers sintered separately (Figure 50). A density gradient is observed in the TiN-AlN layers along the loading axis—the density increases from the center to the free surface of the layer (Figure 51).

**Figure 52:**
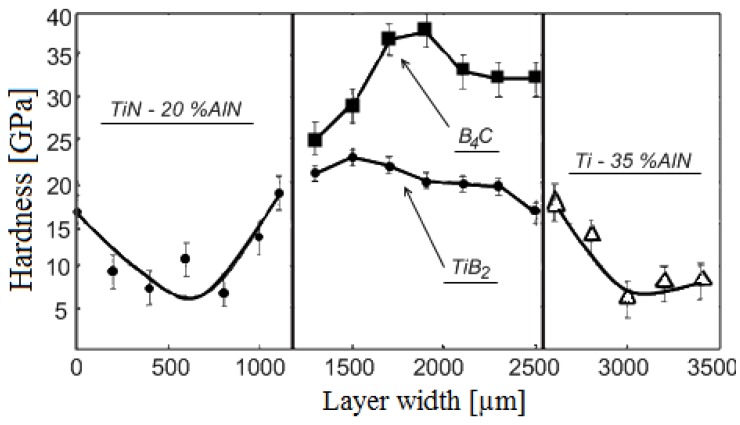
The hardness profile of the composite: TiN-20AlN (1st layer), B_4_C-TiB_2_ (2nd layer), and TiN-35AlN (3rd layer).

Figure 52 shows the hardness profiles of the composite. In the B_4_C–TiB_2_ layer, the density of both coarse-grained phases was measured.

#### 3.2.5. Processing of Cermets

Al_2_O_3_-Fe cermets were sintered by SPS by Kolpakov, Dresvyannokov, Petrova, Doronin and others [97].

Anisimov and Mali of Lavrentiev Institute of Hydrodynamics, Siberian Branch of the Russian Academy of Sciences, Novosibirsk consolidated nanostructured Cu/TiB_2_ composites using electric pulse sintering [132]. The density of the sample containing 90 wt % of Cu was 77%. The particle size of the TiB_2_ inclusions in the Cu matrix ranged from 20 to 40 nm.

Researchers of the Institute of Solid State Chemistry and Mechanochemistry, Siberian Branch of the Russian Academy of Sciences, Novosibirsk studied SPS of Cu-57 vol % TiB_2_ interpenetrating phase composites [118]. The TiB_2_ phase formed agglomerates when melting of the Cu matrix was reached, which is explained by rather poor wettability of TiB_2_ by molten Cu.

Table 8 shows relative density and hardness of the SPS-ed Cu-57 vol % TiB_2_ composites.

**Table 8:** Relative density and hardness of the SPS-ed Cu-57 vol % TiB_2_ composites.

Temperature, °C	Holding time, min	Pressure, MPa	Relative density, %	Hardness, HV
700	5	70	79.4	237
800	5	70	83.0	332
950	0	50	88.8	584
950	5	50	90.0	-
950	30	70	93.9	673

**Figure 53:**
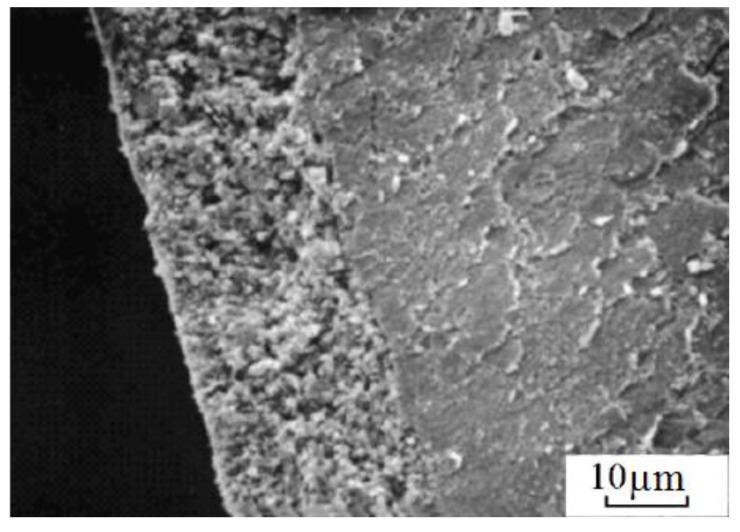
A layer of TiB_2_ revealed on the surface of the Cu/TiB_2_ composite after electrochemical etching.

Having the Cu matrix removed from the surface of the SPS-ed compact by electrochemical etching, a layer of TiB_2_ was revealed confirmed by the XRD phase analysis. A minor phase present after etching was TiBO_3_ likely to form as a result of an electrochemical reaction. The TiB_2_ particles formed a skeleton, which demonstrated that bonding between the particles was established during the SPS (Figure 53).

In another publication, the same group in collaboration with the Institute of Strength Physics and Materials Science, Siberian Branch of the Russian Academy of Sciences, Tomsk developed the processing of the Cu/TiB_2_ composites with different contents of the TiB_2_ phase (10%, 20% and 40%) [117]. Titanium diboride was first synthesized in the Cu matrix from Ti and B powders by the self-propagating high-temperature synthesis. SPS of the Cu-40 wt % TiB_2_ composite powders were sintered in a graphite die at a pressure of 70 MPa varying the maximum temperature in the range of 700–950 °C. The density and hardness of the SPS-ed Cu-40 wt % TiB_2_ composites are shown in Table 9.

**Table 9:** Density and hardness of the SPS-ed Cu-40 wt % TiB_2_.

Temperature, °C	700	950	950
Holding time, min	5	5	30
Density, g/cm^3^	5.1	5.7	6.0
Relative density, %	79.4	89.9	93.9
Hardness, MPa	2320.6	-	6591.5

In Institute of Strength Physics and Materials Science, Siberian Branch of the Russian Academy of Sciences, Tomsk and Institute of Solid State Chemistry and Mechanochemistry, Siberian Brach of the Russian Academy of Sciences, Novosibirsk Stepanova *et al.* [116] studied the microstructure of the Cu-TiB_2_ and Cu-Ni-TiB_2_ composites. The powders that were sintered were prepared by the procedures described above [117]. The authors evaluated several compositions. In the (80% Cu-20% Ni)-10 wt % TiB_2_ composite, the particles of TiB_2_ grew to a lesser extent during sintering than in the Ni-free composite Cu-10 wt % TiB_2_. This difference can be explained by a higher melting temperature of the matrix in the Ni-containing composite. Table 10 shows the mechanical properties of the SPS-ed composites. The optimal composition of the matrix was determined to be 80%:20%. The addition of Ni increased the mechanical strength of the sintered composites.

**Table 10:** Mechanical properties of the SPS-ed Cu-TiB_2_ and Cu-Ni-TiB_2_ composites.

Mechanical properties	Composition					
	10 wt % TiB_2_ + 80Cu + 20Ni	10 wt % TiB_2_ + 60Cu + 40Ni	10 wt % TiB_2_ + 40Cu + 60Ni	10 wt % TiB_2_ + 20Cu + 80Ni	10 wt % TiB_2_ + Cu	20 wt % TiB_2_ + Cu
Relative density, %	95.6	93.7	90.9	91.1	91.8	97.3
Ultimate strength, MPa	1267	1110	1130	1250	510	530
Plasticity, %	~5.5	~1	~1	~1	~4.5	~1
Micrhardness, MPa	4330	6340	6810	7920	2860	3840

In conclusion, sintering of ceramic materials by means of electric current was studied in post-soviet countries: in Kiev, Kharkov, Tomsk, Novosibirsk, Kazan and Makhachkala. The largest number of studies was published during the first decade of the 21st century (2003–2011).

### 3.3. Composites Processed by Electric Current-Assisted Consolidation

**Figure 54:**
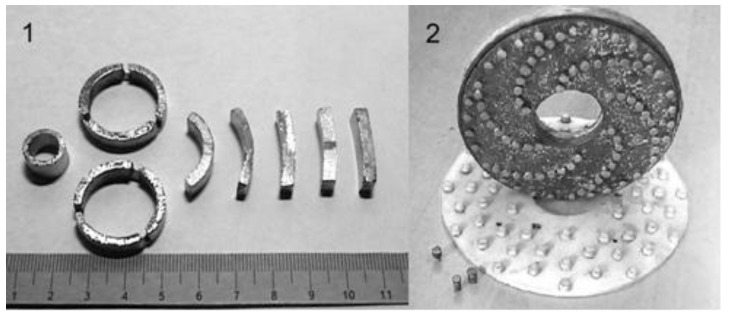
Examples of diamond-metal composite parts sintered by means of electric current: 1—radial segments and drill coronas; and 2—polishing head for natural stone dressing with changeable abrasive elements.

Electric current-assisted sintering of composite materials has been the subject of research in Frantsevich Institute for Problems of Materials Science, National Academy of Sciences of Ukraine and Institute of Pulse Processes and Technologies, National Academy of Sciences of Ukraine since the end of the 1980s. However, other research Institutes have also contributed to the development of this field. Materials studied in this regard can be divided into several groups: Al-graphite and Al-carbon fiber composites [79], B_4_C-TiB_2_ and TiN-AlN composites [87], TiN-TiB_2_ composites [91], Cu-TiB_2_ composites [117], Cu-based and Cu-Ni-based composites [116], Cu-Sn alloys [50,62] and diamond-containing materials [62,120] for applications in stone dressing (Figure 54). The composites were consolidated by high-voltage as well as low-voltage sintering.

In diamond-containing composite materials, Fe, Cu or Co are used as binders. A high-temperature exposure is detrimental to the mechanical properties of diamond-based materials. In order to preserve the mechanical properties of the composites, electric current-assisted consolidation can be a promising solution. In Bakul Institute for Superhard Materials, a sintering technology was developed to sinter these powders at increased pressures.

An electric pulse sintering process of diamond-containing materials was conducted [120]. The pulse duration was 0.5–1.0 ms and the applied pressure was 1–2 GPa. The specific energy evolved was 5–40 kJ/cm^3^. However, the sintering time was too short to achieve efficient sintering of the binder phase and to ensure good mechanical properties.

A model of deformation of diamond-containing composites and their extreme state was developed [133], on the basis of which the optimal concentration of the diamond inclusions was calculated. Experimental studies, however, showed that as the content of the diamond inclusions increase, mechanical properties of the composites degrade, which is related to an increased concentration of defects in the sintered material. The mechanical properties of the initial diamond inclusions can be retained during hot-pressing if the process temperatures correspond to the region of thermodynamic stability of diamond. This, however, does not solve other issues related to composites of this type, namely, poor adhesion of the inclusions to the matrix and a reduced strength of the matrix relative to the conventionally processed matrix metal or alloy containing no inclusions of the reinforcement. These drawbacks can be overcome by low-voltage sintering in a confined volume at a high pressure (up to 0.5 GPa). Preliminary pressing of the powders is conducted in containers made of lithographic stone. Sintering was performed using a unique facility shown in Figure 27 by letting a current pass directly through the sample. Examples of sintered parts manufactured by this method are demonstrated in Figure 54 [120].

Composites based on Fe-Cu-Sn-Ni, Co and M6-14 [133] were prepared. The sintering time was 2 s and the applied pressure was 270 MPa. The properties of the sintered materials with different binders are shown in Table 11.

**Table 11:** Properties of the sintered materials with different binders.

Binder	Resistivity, μOm∙cm	Thermal conductivity, W/m K	Fraction of diamond particles, vol. %	Properties
Fe-Cu-Sn-Ni	22–26	100	25	Density 6.74 g/сm^3^
Co	13–15	170	25	-
M6-14	34–48	87–107	25	Hardness 88–95 HRB

A diamond particle-reinforced composite with a Co binder sintered at temperatures ranging from 670 to 720 °C, which is lower than the optimal temperature, has a relative density of 93.4% while the highest density achievable is 95.5%. The diamond particles play a role of “additional punches” facilitating the densification of the sintered material.

As the pressure increases up to 300 MPa and the voltage decreases from 1 to 0.5–0.625 V, the temperature difference across the volume of the composite decreases to 170 °C, the required sintering time 6.3–10.5 s depending on the voltage.

Sintering of graphite-Al and carbon fabric-Al composites and wear-resistant antifriction composites was studied by Ryabinina. The graphite-Al composites were processed by electric discharge sintering of the MPG-6 graphite powders and PA-4 Al powders using a current density of 370 A/cm^2^, a frequency of alternating current of 2750 Hz, a pressure of 100 MPa and a sintering time of 2 min. The sintered material is of potential interest for the automotive-tractor industry and for electrical applications (contact and sealing rings). Composites with aligned carbon fibers were produced by placing the fibers in a graphite die made of MPG-6 graphite together with an Al foil or an Al powder. The sintered material has a bending strength of 300–350 MPa. Composites “sormite-bronze” and “stellite-bronze” consolidated by electric discharge sintering have the load-bearing capacity 1.5–2 times greater than that of the matrix alone. A composite containing 60 wt % of sormite of a coarse fraction has the highest load-bearing capacity [79].

Mal’tsev studied electric current-assisted rolling to produce ribbons to be used as abrasive and sealing materials. Sintering of a mixture of a 20%Cr-80%Ni alloy, boron nitride, industrial glass and particles of a solid lubricant coated with a layer of Ni 9–12 μm thick (Ni-coated graphite or BN) was performed [18]. Sintering was carried out by applying current pulses of 3800 A with a relative pulse duration of 0.5 and an absolute pulse duration of 0.02 s. The presence of dielectric phases in the mixture influences the results of electric pulse sintering. More dramatic changes in the properties were observed in materials, in which the filler component had a higher microhardness than the matrix.

Composites based on titanium carbides and borides can be used as abrasive materials and materials for cutting tools thanks to their mechanical properties. These powders are sintered by electric discharge sintering at a high temperature and a high pressure [87] and self-propagating high-temperature synthesis (SHS) [91]. Electric discharge sintering offers an easier consolidation procedure; the densities ranging from 64% to 99% can be obtained without the introduction of any additives [91]. The Institute of Solid State Chemistry ad Mechanochemistry, Siberian Branch of the Russian Academy of Science (Novosibirsk) in collaboration with the Institute of Strength Physics and Materials Science, Siberian Branch of the Russian Academy of Science (Tomsk) developed a synthesis route for composite materials “copper–nanosized TiB_2_ particles” [117]. Scientists from Frantsevich Institute for Problems of Materials Science, National Academy of Sciences of Ukraine investigated preparation methods of the B_4_C-TiB_2_ and TiN-AlN composites [87]. Sintering was carried out using ERAN-2/1 facility with the following parameters: a direct current of up to 1.1 kA, an alternating current of high frequency of up to 0.3 kA and an applied pressure of 80 MPa. The TiN-AlN composites were 95% dense, their hardness ranged from 21 to 24 GPa while the fracture toughness was 6.5 MPa·m^1/2^. The B_4_C-TiB_2_ composites were 98% dense with hardness of 35–38 GPa and fracture toughness of ~6.3 MPa·m^1/2^. Microstructure non-uniformities have been found in the TiN-AlN composites explained by the influence of current passing through the samples.

### 3.4. Tooling Materials in the Processes of Electric Current-Assisted Consolidation of Materials

When electric current passes through a conducting powder, the die and the punches as well as the powder itself are involved in complex processes. As electric current passes through the sample and the punches, high temperatures (up to 1000 °C) and pressures (up to 10^6^ kN/m^2^) can develop near their contact. As a result, the material of the punch can experience plastic deformation and, losing its strength, it may fail [77]. Therefore, the material of the punch has to retain strength at elevated temperatures and be highly conductive and wear-resistant. At the same time, the powder that is being sintered should not be prone to sticking to the punch surface [76].

The shape stability of the punches and their interaction with the powder were evaluated [77]. The punches were made of graphite MPG-6, steel 3Cr_2_W_8_V, bronze BrCr_0.7_ and a W-Cu pseudoalloy. Several different powders were sintered: 50% Cu-50% Ni; 80% Ni-20% Mo; 50% W-50% Mo; 70% Mo-30% Cr (wt %). Sintering was performed using a high current density of *j* = 348 ×·10^6^ A/cm^2^ and at a pressure of *P* = 2 ×·10^6^ N/m^2^. It was found that the punches made of BrCr_0.7_ and the W-Cu pseudoalloy deform during the beginning of sintering, experience oxidation and interact with the powder compact. When a graphite lubricant is applied between a Cu-Ni powder compact and a punch made of steel 3Cr_2_W_8_V, the interaction at the interface is prevented, however, the surface becomes contaminated by graphite. The best solution is to coat graphite with a composite coating of the TiN + Si_3_N_4_ composition, which is suitable for multiple sintering cycles as it is stable and does not delaminate [79]. This coating is attractive for sintering of Cu and Fe powders.

The degree of homogeneity of the sintered material depends not only on the total energy input but on the thermal properties of the punches, which can be taken into account by the following function (specific energy input): *E* = (*I*^2^·*t*·*l_cp_*)/(*g·S*) A^2^·s/kg·m, where *I* is the effective current, A; *t* is the sintering time, s; *l_av_* = (*l_1_* + *l_2_*) is an average sample dimension, m; *g* is the sample weight, kg; *S* is the cross-sectional area, m^2^ [77]. During electric discharge sintering of Ni-Mo alloys, the sintering begins at *E* = 5.2·× 10^12^ A^2^·s/kg·m when punches made of BrCr_0.7_ are used while the value of *E* = 1.1·× 10^12^ A^2^·s/kg·m is sufficient when 3Cr_2_W_8_V steel punches are used. For punches made of MPG-6 graphite, the sintering begins at *E* = 0.8·× 10^12^ A^2^·s/kg·m. The differences in the specific energy input are due to the differences in the thermal conductivity of materials of the punches and its temperature dependence. The thermal conductivity of bronze increases with temperature, which leads to heat dissipation, while that of steel does not change with temperature [65,77]. The use of graphite punches is connected with several issues, such as low strength and severe powder sticking to the punch surface, which makes the punches suitable for a single experiment only. These issues can be solved by coating the surface of the electrode by glass ceramics or by impregnating the electrode by salts or high-temperature glass [68]. For sintering of refractory carbide-forming metals, the surface of graphite tooling can be covered by Cr_3_C_2_, TiC and TiN + Si_3_N_4_. However, when Fe, Cu or Cu-Ni powders are sintered, Cr_3_C_2_ and TiC coatings are subject to cracking and delamination [76]. TiB_2_ and ZrB_2_ are not suitable for making the electrodes because of their brittleness and a tendency to catastrophic failure [76]. Problems with electrodes made of steel 3Cr_2_W_8_V are related to non-uniform distribution of carbides and erosion leading to changes in shape [77]. The deformation is usually more severe in the lower punch. Refractory oxygen-free compounds would be promising materials for punches as they only slightly interact with metallic powders, such a Ni-Mo, however, they suffer from increased brittleness. It can be concluded that electrodes can be made of steels similar to 3Cr_2_W_8_V coated by a refractory material to avoid the problem of sticking. A possible interaction of the refractory coating and the sintered material should be taken into account. It was found that alloying of the electrode material with Ti increased the sticking effect when Cu-Sn powders were sintered. Sticking can be minimized upon the introduction of Cu into the material of the punches [58]. When Al powders were sintered using a punch produced by hot-pressing of the TiC + 10% C powder, only minor oxidation effects were observed on the surface of the electrode, its shape remaining unchanged [76].

In electric discharge sintering, the choice of the die material is of great importance. Dies made of Fe-based alloys, Cu, brass, Mo, Cr, Be and WC are used. As thermal and electrical insulation, MgO, Al_2_O_3_, SiO_2_ and Si are suitable candidates. For sintering at high temperatures, graphite, refractory glass and ceramics are used. The resistivity of the die can be tuned by changing the composition of the die material [76].

When Al powders are sintered in a die made of Si_3_N_4_ + MgO, the die experiences severe damage by cracking [76]. Properties of the dies used for electric current-assisted sintering of Cu-20 wt % Sn powders were studied by Svechkov [62]. The reinforcing particles were diamond particles 20–350 μm in size. For Cu-Sn and Cu-Zn powders, dies made of fibrocement were previously used. The punches were made of steel of the 4Cr_5_MoVS type. Due to high friction coefficient between the internal surface of the die and the powder, a convenient die design is an assembly of components (split die), which makes it easier to extract the sintered sample from the die and reduce its wear. Where possible, the dies should have rounded elements to reduce the probability of crack initiation, which is normally higher in the corners. In order to increase the resistance of the electrode and reduce the sticking effect, an electrode design with a sliding needle was suggested [62]. Low-voltage sintering of diamond-containing composites with different matrices was presented by Novikov and Maistrenko [120], who used dies made of lithographic stone.

Overall, the selection of tooling materials is one of the main challenges of electric current-assisted consolidation, as punches and dies experience a combined influence of electric current, temperature and pressure. In order to satisfy the requirements of withstanding load and temperature, the tooling materials have to be of composite structure or coatings need to be applied to protect the main material [76].

## 4. Modeling of Electric Current-Assisted Consolidation of Powder Materials 

Russian scientists and scientists from post-soviet countries have directed substantial attention to the development of theoretical models describing the processes of sintering of powder materials by electric current-assisted methods. One third of the citations in this review are papers dealing with theoretical investigation of these sintering processes.

The efficiency of the electric current-assisted methods of consolidation depends on the collective influence of thermal, electrical and mechanical impacts on the material. Spatial and temporal inhomogeneities on micro- and macroscales are characteristics of the physical processes occurring during sintering of materials by means of electric current. The most widely considered problems in the theoretical studies, as can be concluded on the basis of this review, are those directed at finding the general laws, the temperature distribution fields [27,119,134,135,136], electrical resistance of the sintered material as well as current density and voltage [14,15,37,39,46,137]. Several studies have been concerned with the solution of the thermo-electric problem [10,33,138]. The solution of this problem is of critical importance to the mass transfer processes and is related to the properties of the sintered material. Theoretical studies are aimed at determining the optimal consolidation parameters of powder materials to ensure the formation of materials of targeted properties.

Macroscopic modeling of electric discharge sintering was done by Balankin *et al.* [10] by numerical solution of the heat conduction equation for a consolidated material taking into account the Joule heat. The boundary conditions determined the heat exchange of the sintered sample with the punches. The current density was determined by the quasi-steady-state equation of current flow taking into account the dependence of the resistivity on temperature. In Frantsevich Institute for Problems of Materials Science, National Academy of Sciences of Ukraine, Raichenko *et al.* [134,135,139] considered a similar problem and studied the influence of electric current on the processes at the interface between the powder compact and the punch. Denoting the temperature of the powder compact as *T_1_* and the temperature of the punches as *T_2_*, a system of equations can be composed (1), which determines the temperature distribution in the sample (*i* = 1) and punches (*i* = 2) along the vertical Z-axis. The heat dissipation in the radial direction is ignored. The system of Equations (1) is solved for a set of boundary and initial conditions (2).

(1) $$\frac{\partial T_{i}}{\partial t}=\frac{\lambda_{i}}{c_{pi}\gamma_{i}}\frac{\partial^{2}T_{i}}{\partial x^{2}}+\frac{\rho_{i}{\vec{j}}^{2}}{c_{p}\gamma_{2}},\;i=1,\;(0\leq x\leq l_{1});\;i=2,\;(l_{1}\leq x\leq l_{1}+l_{2});$$

(2) $$\begin{array}{l} T_{1}(x,0)=T_{2}(x,0)=T_{0};\\ {\frac{\partial T_{1}}{\partial x}|}_{x=0}=0;\\ {\lambda_{1}\frac{\partial T_{1}}{\partial x}|}_{x=l_{1}}={\lambda_{2}\frac{\partial T_{2}}{\partial x}|}_{x=l_{1}};\\ T(l_{1},t)=T_{2}(l_{1},t);\\ T_{2}(l,t)=T_{0}, \end{array}$$

where variables with *i* = 1 relate to the material of the sample while variables with *i* = 2 relate to the material of the punches; *T_i_* is the temperature, *T*_0_ is the initial temperature of the system, *t* is the time of consolidation, *c_pi_* is the heat capacity, γ*_i_* is the density, *λ_i_* is thermal conductivity, *x* is the coordinate taken from the center of a cylindrical sample, *ρ_i_* is the resistivity, *j* is the current density, *l_i_* is the geometrical parameter.

As a result of calculations performed analytically, the temperature field in the sintered sample and punches was determined as a function of the sintering time. The heating and cooling rates of the sample as functions of the sample’s porosity, emissivity factor of the sample surface, the dimensions of the sample and properties of the sintered material were calculated by Belyavin *et al.* [27], who considered the thermal problem taking into account the theoretical dependence of the electrical resistance on the temperature. The distribution of electrical resistance and strains in the powder compact as well as pressure created by magnetic forces along the radius of a cylindrical powder compact was described by Raichenko [137].

The calculation of the temperature field during sintering by electric current by solving the heat conduction equation with a set of initial and boundary conditions was also presented by Belousov *et al.* [119] for non-conducting materials.

The skin and pinch-effects during electric discharge sintering were described by Kaptsevich *et al.* [31], Vityaz *et al.* [43] and other scientists from the Institute of Powder Metallurgy, Belarus.

Mass transport through the diffusion processes during electric discharge sintering was presented by Raichenko [2,29] in two monographs. The diffusion equation was solved for liquid-phase and solid-state contacts for the case of one and the same material as well as for the case of two different materials. A theoretical analysis of the dependence of the rate of the diffusion processes on the defect structure of the crystalline lattice of the sintered materials was done by Andrushchik *et al.* [140]. Theoretical research on the effect of the inhomogeneous distribution of temperature on the diffusion processes and sintering of magnetic materials was reviewed by Kornyushin [138].

A method of semi-phenomenological determination of the macroscopic characteristics of the powder compacts obtained by electric discharge sintering using the particle spatial distribution function was presented by Alexandrov *et al.* [32]. The resultant distribution function depends on the correlation function, which correlated the diameters of two particles. The correlation function has a rather simple analytical form and allows describing correlations in the particle arrangements, which were studied experimentally. An algorithm has been suggested to use this distribution function for real powder materials. The results confirm the existence of inter-particle correlations and indicate that a random packing of particles in a porous medium is governed by the laws of statistical physics.

Popov and Grigoryev [141] developed a model that allows the deformation mechanism typical for the current temperature of the sample, its porosity and externally applied pressure to be determined.

Electric pulse sintering was studied by Grigoryev [53,142,143] (applied-research laboratory No. 709 of Moscow Engineering Physics Institute). The interrelation of the electrodynamic, thermal and deformation processes during sintering was considered. Relationships between the characteristic time scales of the electric pulse sintering were established. Belyavin *et al.* [33] of the Institute of Powder Metallurgy, Belarus derived equations to calculate the heat evolution, the efficiency of the electric circuit for electric pulse sintering, the resistance of the powder compact and the temperature in the contact zones between the particles. An algorithm was developed to calculate the optimal technological parameters of electric pulse sintering.

Using a system of equations describing the process of electric pulse sintering and comprising the mass and momentum conservation laws and the electrodynamic equations for a powder compact of conducting material, Olevsky and Grigoryev [136] conducted a multi-scale analysis of the temperature distribution during electric pulse sintering. The process of densification was described using a viscoplastic model. The analysis allowed heat evolution in the contact zones and macro- and microscopic temperature field in the contact zone between the powder particles to be determined. The characteristic time scales of the thermal processes involved in electric pulse sintering were estimated. The modeling results point at a possibility of intensive local heating of the inter-particle contacts under certain consolidation parameters.

A review of macroscopic models of pressing enabling determination of the density and porosity of the powder compact as functions of applied pressure was presented by Zhdanovich [144]. A model of electric current-assisted sintering during pressing was outlined by Mal’tsev [14,15], in which an equation was given to calculate the required voltage applied to the rollers during the process of powder rolling; the influence of the applied voltage on the consolidation process was also discussed.

Vityaz *et al*.[37], Belyavin *et al*.[38] investigated the formation laws of the inter-particle contacts during electric pulse sintering. A theoretical description of the formation mechanisms of the inter-particle contacts was presented that allows the prediction of the microstructure and mechanical properties of the sintered materials. An equation of the contact growth kinetics was derived from an energy balance equation of a single inter-particle contact. This equation is simultaneous with relationships between the structural parameters of the powder material and its porosity and mechanical strength, and makes it possible to calculate the changes in these properties occurring during electric pulse sintering. It was shown that the mechanical strength and porosity establish during the first half-cycle of voltage. Using the conservation laws of mass, momentum and energy simultaneously with the electrodynamic equations, the temperature field was calculated in the inter-particle contact zone between two metallic particles for the case of deposition of coatings by electric pulse sintering [54].

One of the possible mechanisms of contact area growth is a dislocation-based mechanism. Boiko and Klinchuck [145] developed a dislocation model of electric pulse sintering. The dependencies of the radius of the contact spot on the current amplitude and the size of the powder particle obtained from the model are in agreement with the experimental data.

Meshkov *et al.* [146] suggest that the major contribution to densification, stems from the electrical conductivity changes of the sintered material. A model was developed describing the conductivity of the powder column solving the Laplace equation solved for two metallic spheres in contact with each other. The results of the calculations were used to develop a structured scheme of consecutive selection of the electric discharge sintering parameters. A model of conductivity variation in the contact zone during electric discharge sintering was presented by Pakhomov *et al.* [147]. Using the heat conduction equation and the Joule-Lenz law, an equation was obtained for the evolution of the radius of the contact zone during sintering. Plots were obtained showing dependence of the resistance of the contact zone on the characteristics of the pulsed current and current distribution in the sample.

In this part of the article, the main directions of theoretical investigations of the processes of electric current-assisted consolidation conducted in Russia and post-soviet countries were briefly reviewed. The majority of publications were devoted to electric discharge and electric pulse sintering. The main tasks of theoretical modeling included finding a solution of a consistent thermo-electric problem and determining electrical resistance (for conducting materials) as a function of time, temperature, macroscopic geometry changes of the sample and microscopic evolution of the contact zones. Models were developed describing the temperature distribution in the contact zones. The developed models and performed calculations are useful to optimize the sintering process by choosing the sintering parameters for certain material and processes.

## 5. Conclusions

In this review, we have made an attempt to cover the majority of articles published in the USSR and post-soviet countries on consolidation of powder materials with the help of electric current either passing through the sintered material directly or through a conductive die. Statistical data are also presented showing activities of different research organizations in this field during different time periods. Electric current-assisted sintering methods are described and grouped as low- and high-voltage techniques. The experimental approaches and related equipment as well as the main experimental results are presented. It can be concluded that the studies in the area of electric current-assisted sintering of powder materials using electric currents directly passing through the sample were actively developing throughout the whole period considered. Several sintering set-ups were designed; some of them were modernized and were used until recently.

A large amount of work was done to optimize the sintering conditions for different material systems. Several authors performed a comparative analysis of the sintering results produced on one and the same material consolidated by different sintering techniques. The challenges of consolidation of different materials (metals, alloys, ceramics and composites) are outlined and possible solutions suggested by the authors are discussed. Further, the properties of the consolidated materials as well as their potential applications are demonstrated.

The problem of choosing the tooling material is considered. Overall, the review indicates that the selection of tooling materials is one of the main challenges of electric current-assisted consolidation, as punches and dies experience a combined influence of electric current, temperature and pressure. In order to satisfy the requirements of withstanding load and temperature, the tooling materials may need to be of composite structure or coatings need to be applied to protect the main material.

General modeling approaches developed for the processes of consolidation of powder materials under the action of electromagnetic fields are described. The majority of publications were devoted to electric discharge and electric pulse sintering. The main tasks of theoretical modeling included finding a solution of a consistent thermo-electric problem and determining electrical resistance (for conducting materials) as a function of time, temperature, macroscopic geometry changes of the sample and microscopic evolution of the contact zones. Models were developed describing the temperature distribution in the contact zones. The developed models and performed calculations are useful to optimize the sintering process by choosing the sintering parameters for certain material and processes.
